# Moving from two- to multi-way interactions among binary risk factors on the additive scale

**DOI:** 10.1080/24709360.2020.1850171

**Published:** 2020-12-03

**Authors:** Michail Katsoulis, Manuel Gomes, Christina Bamia

**Affiliations:** a Institute of Health Informatics, University College London, London, UK; b Hellenic Health Foundation, Athens, Greece; c Institute of Epidemiology & Health Care, University College London, London, UK; d Department of Hygiene, Epidemiology and Medical Statistics, Medical School, National and Kapodistrian University of Athens, Athens, Greece

**Keywords:** RERI, additive, interaction, multi-way, relative excess risk due to interaction

## Abstract

Many studies have focused on investigating deviations from additive interaction of two dichotomous risk factors on a binary outcome. There is, however, a gap in the literature with respect to interactions on the additive scale of >2 risk factors. In this paper, we present an approach for examining deviations from additive interaction among three or more binary exposures. The relative excess risk due to interaction (RERI) is used as measure of additive interaction. First, we concentrate on three risk factors – we propose to decompose the total RERI to: the RERI owned to the joint presence of all three risk factors and the RERI of any two risk factors, given that the third is absent. We then extend this approach, to >3 binary risk factors. For illustration, we use a sample from data from the Greek EPIC cohort and we investigate the association with overall mortality of Mediterranean diet, body mass index , and smoking. Our formulae enable better interpretability of any evidence for deviations from additivity owned to more than two risk factors and provide simple ways of communicating such results from a public health perspective by attributing any excess relative risk to specific combinations of these factors.

**Abbreviations:** BMI: Body Mass Index; ERR: excess relative risk; EPIC: European Prospective Investigation into Cancer and nutrition; MD: Mediterranean diet; RERI: relative excess risk due to interaction; RR: relative risk; TotRERI: total relative excess risk due to interaction

## 1. Introduction

Four decades ago, Rothman stated that, as more than one risk factors are eventually established for the etiology of a specific health outcome, epidemiologists will need to pay more attention to the issue of interaction (synergy or antagonism) between these factors [1]. This is particularly relevant in the field of genetic epidemiology, as scientists focus on the study of thousands of genes and of their interactions with environmental factors [2–3].

Measuring interaction on the additive scale is more important from a public health perspective [4–7], because, in this context, two risk factors are independent, when the number of disease cases is not dependent on the extent to which these factors act together [5]. If, for example, the number (or rate) of hospitalizations for a disease when individuals are exposed into two risk factors is greater than the sum of hospitalizations for this disease of the people exposed only to one of these factors, then the public health services would be challenged to carry extra weight due to this interaction, which is measured as a deviation from additivity of the effects of these factors.

Nevertheless, the usual practice has been to refer to statistical interaction when studying interaction between risk factors [7]. Under this concept, interaction is measured on either additive or multiplicative scale, depending solely on the form of the underlying model used, rather than on a-priori consideration for the expected type of associations between these exposures and the outcome.

The deviation from additivity of the effects between two variables has been proposed by Rothman [8] and further explored by others [2–3,9–22]. Surprisingly, the study of additive interaction of >2 factors has not been studied adequately, even if it would enable better understanding of the joint action of many factors for the development of a specific disease. This may have occurred because conceptualizing the features of multi-way interaction is challenging (e.g. a modification of an interaction between two variables by a third variable is not easy to understand and sometimes the rationale for assessing this effect is not there). Apart from the studies related to multi-way interaction in the sufficient-cause framework [23–25], to the best of our knowledge, there are only three relevant publications that have focused only on practical illustration of deviation from additivity of the effects of three risk factors [26–28].

In this paper, we aim to fill this gap in the literature and provide useful tools for researchers who wish to focus on the joint action of >2 binary factors. We highlight the questions of interest in the study of joint action of three factors, we give answers by introducing useful indexes for additive interaction, accompanied by the corresponding recommendation for the conduction these of analyses, and we then extend the methods to >3 factors. We illustrate our theoretical arguments using data from the Greek-EPIC study and we provide an easy-to-use code in Stata for the implementation of these methods.

## 2. Methods

### 2.1. Definitions

Consider *n* dichotomous *X_i_*, *i* =1,  … , *n* variables as risk factors for a disease D with *X_i_*_ _= (0,1). Let D+ and D− denote the presence/absence of D, and *X_i_+*, *X_i_*−, the presence (*X_i_* = 1) or absence (*X_i_* = 0) of *X_i_*. The relative risk of D+ for any combination of the presence or absence of *X*_1_, *X*_2_, … *X_n_*, as compared to their absence is denoted by

$\text{R}{\text{R}}_{X_{1}\#X_{2}\#\ldots X_{n}\#}$, where # = +/− and the corresponding excess relative risk by $\text{ER}{\text{R}}_{X_{1}\#X_{2}\#\ldots X_{n}\#}$ with

$$\begin{array}{l} \text{ER}{\text{R}}_{X_{1}\#X_{2}\#\ldots X_{n}\#}=\text{R}{\text{R}}_{X_{1}\#X_{2}\#\ldots X_{n}\#}-\text{R}{\text{R}}_{X_{1}-X_{2}-\ldots X_{n}-},\text{ i}.\text{e}.\\ \text{ER}{\text{R}}_{X_{1}\#X_{2}\#\ldots X_{n}\#}=\text{R}{\text{R}}_{X_{1}\#X_{2}\#\ldots X_{n}\#}-1 \end{array}$$

For the investigation of any deviation from additivity of the effects of two risk factors, we focus on the contrast between

$$\text{R}{\text{R}}_{X_{1}+X_{2}+}-\text{R}{\text{R}}_{X_{1}-X_{2}-}\text{vs}(\text{R}{\text{R}}_{X_{1}+X_{2}-}-\text{R}{\text{R}}_{X_{1}-X_{2}-})+(\text{R}{\text{R}}_{X_{1}-X_{2}+}-\text{R}{\text{R}}_{X_{1}-X_{2}-})$$

i.e. the excess risk from the situation when two risk factors act jointly versus the extra risk of the occasions that each of them acts separately

So a measure for additive interaction would be the relative excess risk due to interaction RERI_2_(*X*_1_,*X*_2_)

$$\begin{array}{rl} \text{RER}{\text{I}}_{2}(X_{1},X_{2}) & =\text{ER}{\text{R}}_{X_{1}+X_{2}+}-\text{ER}{\text{R}}_{X_{1}+X_{2}-\;}-\text{ ER}{\text{R}}_{X_{1}-X_{2}+}\\ & =(\text{R}{\text{R}}_{X_{1}+X_{2}+}-\text{R}{\text{R}}_{X_{1}-X_{2}-})-(\text{R}{\text{R}}_{X_{1}+X_{2}-}-\text{R}{\text{R}}_{X_{1}-X_{2}-})-(\text{R}{\text{R}}_{X_{1}-X_{2}+}\\ & \;-\text{R}{\text{R}}_{X_{1}-X_{2}-})\\ & =\text{R}{\text{R}}_{X_{1}+X_{2}+}-\text{R}{\text{R}}_{X_{1}+X_{2}-}-\text{R}{\text{R}}_{X_{1}-X_{2}+}+1 \end{array}$$

which indicates whether the effect of two risk factors that act jointly is greater (RERI_2_ > 0), equal (RERI_2_ = 0) or lower (RERI_2_<0) than the sum of their individual effect (super-additive, additive, or sub-additive effects, respectively).

It is crucial to highlight that the factors cannot be protective, because the calculation of additive interaction will be wrong, as a relative risk is between 0 and 1 for a protective factor, while it can be from 1 to infinity for a risk factor. Imagine two drugs with additive effects (RERI_2_ = 0) on CVD, each of those reducing the CVD risk by 75% (i.e. RR_10_, RR_01_ = 0.25). We cannot use the RERI_2_ index, because we would calculate that RR_11_ is negative (RR_11_ = –0.5)! Instead, we should recode these factors into risk (i.e. the effect of not taking the drugs) and apply the calculations (see [9] and Appendix, Section B and D).

Additionally, if one wants to focus on the multiplicative interaction of two risk factors, then the contrast of interest would be

$$\text{R}{\text{R}}_{X_{1}+X_{2}+}\;\;\text{vs}\;\;\;\text{R}{\text{R}}_{X_{1}+X_{2}-}*\text{R}{\text{R}}_{X_{1}-X_{2}+}$$

i.e. the comparison of the relative risk when two risk factors act jointly versus the multiplication of the risk of the occasions that each of them acts separately

The index of multiplicative interaction would be

$$I_{2}=\frac{\text{R}{\text{R}}_{X_{1}+X_{2}+}}{\text{R}{\text{R}}_{X_{1}+X_{2}-}*\text{R}{\text{R}}_{X_{1}-X_{2}+}}$$

For more details, see ref [16].

In Appendix (Section E), we show (i) that multiplicative or super-multiplicative effects imply super-additive effects and (ii) that additive or sub-additive effects imply sub-multiplicative effects for two-way interactions.

#### 2.1.1. From the two to the three-way interaction on the additive scale

Imagine now that the question of interest is whether three risk factors ‘interact’ on the additive scale. How should we face that problem?

The first answer we should give would be an extension of the previous methods for the construction of RERI_2_. Now, we should take into account the extra risk due to the joint presence of the three risk factors and compare it with the sum of the excess risks caused by each risk factor separately, i.e.

$$\begin{array}{rl} & \left(\text{R}{\text{R}}_{X_{1}+X_{2}+X_{3}+}-\text{R}{\text{R}}_{X_{1}-X_{2}-X_{3}-})\text{ vs }\;(\text{R}{\text{R}}_{X_{1}+X_{2}-X_{3}-}-\text{R}{\text{R}}_{X_{1}-X_{2}-X_{3}-}\right)\\ & \;+(\text{R}{\text{R}}_{X_{1}-X_{2}+X_{3}-}-\text{R}{\text{R}}_{X_{1}-X_{2}-X_{3}-})\\ & \;+(\text{R}{\text{R}}_{X_{1}-X_{2}-X_{3}+}-\text{R}{\text{R}}_{X_{1}-X_{2}-X_{3}-})\; \end{array}$$

In other words, we should extend the RERI definition to three risk factors *X*_1_, *X*_2_ and *X*_3_ and calculate the total relative excess risk due to interaction (TotRERI_3_),

(1) $$\begin{array}{rl} & \text{TotRER}{\text{I}}_{3}(X_{1},X_{2},X_{3})\\ & \;=(\text{R}{\text{R}}_{X_{1}+X_{2}+X_{3}+}-\text{R}{\text{R}}_{X_{1}-X_{2}-X_{3}-})-(\text{R}{\text{R}}_{X_{1}+X_{2}-X_{3}-}-\text{R}{\text{R}}_{X_{1}-X_{2}-X_{3}-})\\ & \;-(\text{R}{\text{R}}_{X_{1}-X_{2}+X_{3}-}-\text{R}{\text{R}}_{X_{1}-X_{2}-X_{3}-})-(\text{R}{\text{R}}_{X_{1}-X_{2}-X_{3}+}-\text{R}{\text{R}}_{X_{1}-X_{2}-X_{3}-})\\ & \;=\text{R}{\text{R}}_{X_{1}+X_{2}+X_{3}+}-\text{R}{\text{R}}_{X_{1}+X_{2}-X_{3}-}-\text{R}{\text{R}}_{X_{1}-X_{2}+X_{3}-}-\text{R}{\text{R}}_{X_{1}-X_{2}-X_{3}+}+2 \end{array}$$

The total relative excess risk due to interaction (TotRERI_3_) is calculated by comparing the joint effect of three risk factors to the situation when each one acts separately. It allows us to understand whether these variables have super-additive, additive, or sub-additive effects.

The next issue that we should wonder is about the index for 3-way additive interaction, beyond two-way interactions. The super/sub additivity of the effects of three risk factors (1) is due either to the three-way interaction (RERI_3_) of the three risk factors, or to the two-way interaction of the two risk factors, when the third is absent. To calculate RERI_3_, one needs to subtract RERI_2_(*X*_1_, *X*_2_ | *X*_3_ = 0), RERI_2_ (*X*_1_, *X*_3_ | *X*_2_ = 0) and RERI_2_ (*X*_2_, *X*_3_ | *X*_1_ = 0) from TotRERI_3_, i.e.

$$\begin{array}{rl} \text{RER}{\text{I}}_{3}(X_{1},X_{2},X_{3}) & =\text{TotRER}{\text{I}}_{3}(X_{1},X_{2},X_{3})-\text{RER}{\text{I}}_{2}(X_{1},X_{2}|X_{3}=0)\\ & \;-\text{RER}{\text{I}}_{2}(X_{1},X_{3}|X_{2}=0)-\text{RER}{\text{I}}_{2}(X_{2},X_{3}|X_{1}=0) \end{array}$$

The relative excess risk due to interaction is the measure of the three-way interaction. This index indicates whether there is positive/negative three-way interaction on the additive scale, which is explicitly due to the joint presence of all three factors, in other words, measures the three-way interaction, beyond the possible two-way interactions.

Additionally, $\text{TotRER}{\text{I}}_{3}(X_{1},X_{2},X_{3})$ expresses the sum of the three-way interaction and all two-way interactions and that is,

(2) $$\begin{array}{rl} \text{TotRER}{\text{I}}_{3}(X_{1},X_{2},X_{3}) & =\text{RER}{\text{I}}_{3}(X_{1},X_{2},X_{3})+\text{RER}{\text{I}}_{2}(X_{1},X_{2}|X_{3}=0)\\ & \;+\text{RER}{\text{I}}_{2}(X_{1},X_{3}|X_{2}=0)+\text{RER}{\text{I}}_{2}(X_{2},X_{3}|X_{1}=0) \end{array}$$

Of note, TotRERI_3_ may be zero as the result of two-way interactions that cancel out with three-way interaction.

Moreover, to calculate the three-way interaction $\text{(RER}{\text{I}}_{3}(X_{1},X_{2},X_{3}))$, we have to combine (1) and (2) (see Appendix, section A) to estimate

(3) $$\begin{array}{rl} \text{RER}{\text{I}}_{3}(X_{1},X_{2},X_{3}) & =\text{R}{\text{R}}_{X_{1}+X_{2}+X_{3}+}-\text{R}{\text{R}}_{X_{1}+X_{2}+X_{3}-}-\text{R}{\text{R}}_{X_{1}+X_{2}-X_{3}+}\\ & \;-\text{R}{\text{R}}_{X_{1}-X_{2}+X_{3}+}+\text{R}{\text{R}}_{X_{1}+X_{2}-X_{3}-}+\text{R}{\text{R}}_{X_{1}-X_{2}+X_{3}-}\\ & \;+\text{R}{\text{R}}_{X_{1}-X_{2}-X_{3}+}-\text{R}{\text{R}}_{X_{1}-X_{2}-X_{3}-} \end{array}$$

Finally, we note that the three-way interaction (see Equation (3)) reflects the contrast of interactions between two variables over the strata of a third. We show in Appendix (Section A) that the corresponding formulae are

(4) $$\begin{array}{r} \text{RER}{\text{I}}_{3}(X_{1},X_{2},X_{3})=(\text{RER}{\text{I}}_{2}(X_{j},X_{k}|X_{\text{l}}=1)*\text{R}{\text{R}}_{X_{j}-X_{k}-X_{l}+})-\text{RER}{\text{I}}_{2}(X_{j},X_{k}|X_{l}=0) \end{array}$$

because

$$\text{RERI}(X_{j},X_{k}|X_{l}=1)=\frac{\left(\text{R}{\text{R}}_{X_{j}+X_{k}+X_{l}+}-\text{R}{\text{R}}_{X_{j}+X_{k}-X_{l}+}-\text{R}{\text{R}}_{X_{j}-X_{k}+X_{l}+}+\text{R}{\text{R}}_{X_{j}-X_{k}-X_{l}+}\right)}{\text{R}{\text{R}}_{X_{j}-X_{k}-X_{l}+}}$$

given that $\text{R}{\text{R}}_{X_{j}-X_{k}-X_{l}-}$ is the reference relative risk.

where *j*, *k*, *l* = (1, 2, 3) and *j*≠*k*, *j*≠*l*, *k*≠*l*.

The two-way interactions, given the third factor is absent $\text{(RER}{\text{I}}_{2}(X_{1},X_{2}|X_{3}=0)$, $\text{RER}{\text{I}}_{2}(X_{1},X_{3}|X_{2}=0)$ and $\text{RER}{\text{I}}_{2}(X_{2},X_{3}|X_{1}=0)$, as well as the corresponding interactions when the third risk factor is present $\text{(RER}{\text{I}}_{2}(X_{1},X_{2}|X_{3}=1)$, $\text{RER}{\text{I}}_{2}(X_{1},X_{3}|X_{2}=1)$ and $\text{RER}{\text{I}}_{2}(X_{2},X_{3}|X_{1}=1)$ are important measures in the study of joint effects of three factors. They are very helpful in better specifying under which conditions two of the three factors interact. In the classic framework of two-way interactions, researchers report a specific value for RERI between two variables *X*_1_ and *X*_2_. However, this value may not be constant across the strata of a third factor *X*_3_ and to check for that issue (which was named ‘the uniqueness problem’ by Skrondal [12]), we can calculate $\text{RER}{\text{I}}_{2}(X_{1},X_{2}|X_{3}=0)$ and $\text{RER}{\text{I}}_{2}(X_{1},X_{2}|X_{3}=1)$.

In the Appendix (section B), we show how to calculate the formulae of all these indexes for additive interaction in the presence of three risk factors (both two- and three-way interactions), when applying Cox regression. The formulae are the same when using logistic regression as well. Finally, we provide user-friendly Stata code that would be useful for researchers who wish to implement these methods and calculate all possible two- and three-way interactions (Appendix, section B).

#### 2.1.2. From the three to the multi-way interaction on the additive scale

When studying the multi-way interaction of n risk factors *X*_1_, *X*_2_, … ,*X_n_*, on the additive scale, we can calculate the total relative excess risk due to interaction $\text{(TotRER}{\text{I}}_{n})$, as a generalization of Equation (1). $\text{TotRER}{\text{I}}_{n}$ expresses the contrast of the excess risk from the situation when all risk factors act jointly versus the extra risk of the occasion that each of them acts separately, i.e.

When studying the multi-way interaction of n risk factors *X*_1_, *X*_2_ … , *X_n_*, on the additive scale, the comparison of interest is

$$\begin{array}{rl} & (\text{R}{\text{R}}_{X_{1}+X_{2}+\ldots X_{n}+}-\text{R}{\text{R}}_{X_{1}-X_{2}-\ldots X_{n}-})\text{ vs}\;(\text{R}{\text{R}}_{X_{1}+X_{2}-\ldots X_{n}-}-\text{R}{\text{R}}_{X_{1}-X_{2}-\ldots X_{n}-})\;\\ & \;+(\text{R}{\text{R}}_{X_{1}-X_{2}+\ldots X_{n}-}-\text{R}{\text{R}}_{X_{1}-X_{2}-\ldots X_{n}-})\;\\ & \;+\ldots \\ & \;+(\text{R}{\text{R}}_{X_{1}-X_{2}-\ldots X_{n}+}-\text{R}{\text{R}}_{X_{1}-X_{2}-\ldots X_{n}-}) \end{array}$$

The difference from this comparison should correspond to the total relative excess risk due to interaction, that is the sum of the n-way interaction and all the (*n*-1)-, (*n*-2)-, … , two-way interactions of these risk factors. In other words, we have that,

(5) $$\begin{array}{rl} \text{TotRER}{\text{I}}_{n}(X_{1},X_{2},\ldots ,X_{n}) & =\text{ER}{\text{R}}_{X_{1}+X_{2}+\ldots X_{n}+}-\text{ER}{\text{R}}_{X_{1}+X_{2}-\ldots X_{n}-}\\ & \;-\text{ER}{\text{R}}_{X_{1}-X_{2}+\ldots X_{n}-}-\ldots -\text{ER}{\text{R}}_{X_{1}-X_{2}-\ldots X_{n}+} \end{array}$$

Given that $\text{TotRER}{\text{I}}_{n}$ is attributed to all potential interactions between the n variables, in other words it can be expressed as the sum of the *n*-way interaction and all the (*n*-1)-, (*n*-2)-, … , two-way interactions (see Appendix, section C), i.e.

$$\begin{array}{rl} \text{TotRER}{\text{I}}_{n}(X_{1},X_{2},\ldots ,X_{n}) & =\text{RER}{\text{I}}_{n}(X_{1},X_{2},\ldots ,X_{n})\\ & \;+\sum_{\left(\begin{array}{c} n\\ n-1 \end{array}\right)}\text{RER}{\text{I}}_{n-1}(X_{1},X_{2},\ldots ,X_{n}|1\;\text{of the}\;X_{i}=0)\\ & \;+\sum_{\left(\begin{array}{c} n\\ n-2 \end{array}\right)}\text{RER}{\text{I}}_{n-2}(X_{1},X_{2},\ldots ,X_{n}|2\;\text{of the}\;X_{i}=0)\\ & \;\ldots \\ & \;+\sum_{\left(\begin{array}{c} n\\ 2 \end{array}\right)}\text{RER}{\text{I}}_{2}(X_{1},X_{2},\ldots ,X_{n}|(n-2)\;\text{of the}\;X_{i}=0) \end{array}$$

If we want to compute the *n*-way additive interaction RERI_n_, without the contribution of all lower order interactions, then for 1≤*i *≤ *n* and 1≤*k* ≤ *n*, we let

$\text{R}{\text{R}}_{\left(\text{k}\right)}$ equal to $\text{R}{\text{R}}_{\text{k of the }{{\text{X}}_{\text{i}}}^{\prime }\text{s}=1,\text{ n}-\text{ k of the }{{\text{X}}_{\text{i}}}^{\prime }\text{s}=0}$

We show in the Appendix (section C) that, $\text{RER}{\text{I}}_{\text{n}}({\text{X}}_{1},{\text{X}}_{2},\ldots ,{\text{X}}_{\text{n}})$ for *n* ≥ 2, is:

(6) $$\begin{array}{rl} \text{RER}{\text{I}}_{n}(X_{1},X_{2},\ldots ,X_{n}) & =\text{R}{\text{R}}_{\left(n\right)}\\ & \;-\sum_{\left(\begin{array}{c} n\\ n-1 \end{array}\right)}\text{R}{\text{R}}_{\left(n-1\right)}\\ & \;+\sum_{\left(\begin{array}{c} n\\ n-2 \end{array}\right)}\text{R}{\text{R}}_{\left(n-2\right)}\\ & \;\ldots \\ & \;+(-1{)}^{n}*\sum_{\left(\begin{array}{c} n\\ 0 \end{array}\right)}\text{R}{\text{R}}_{\left(0\right)} \end{array}$$

Of note that the last line can be written as $(-1{)}^{n}$, once $\left(\begin{array}{c} n\\ 0 \end{array}\right)=1$ and $\text{R}{\text{R}}_{\left(0\right)}=1.$

$\text{RER}{\text{I}}_{n}(X_{1},X_{2},\ldots ,X_{n})$ in (6) expresses the relative excess risk due to interaction of the n risk factors exclusively, without accounting for all the lower order additive interactions of these risk factors [i.e. extension of (4)].

Additionally, by extending Equation (4) in multi-way interaction, we additionally show in the Appendix (section C) that $\text{RER}{\text{I}}_{n}$ can be written in terms of any two of the lower order (n-1) interactions, more specifically

(7) $$\begin{array}{rl} \text{RER}{\text{I}}_{n}(X_{1},X_{2},\ldots ,X_{n}) & =(\text{RER}{\text{I}}_{n-1}(X_{1},X_{2},\ldots ,X_{n}|X_{i}=1)\\ & \;*\text{R}{\text{R}}_{X_{1}-X_{2}-\ldots X_{i+1}-X_{i}+X_{i+1}-\ldots X_{i+1}-\ldots X_{n}-})\\ & \;-\text{RER}{\text{I}}_{n-1}(X_{1},X_{2},\ldots ,X_{n}|X_{i}=0) \end{array}$$

In Appendix (section D), we give the corresponding suggestions and recommendations for researchers who want to implement analysis for multi-way interactions in detail. Moreover, in Appendix (Section E), we show (i) that multiplicative or super-multiplicative effects imply super-additive effects and (ii) that additive or sub-additive effects imply sub-multiplicative effects for *n*-way interactions as well.

## 3. Worked example

To illustrate the formulae derived in the previous sections, we have used data from adult women participating in the Greek-EPIC study [29–30] to study the joint effects of low adherence to Mediterranean Diet (MD), obesity, and smoking status on mortality. We applied survival analysis with Cox regression using as endpoint death from any cause. Levels of the indicated risk factors denoting potentially increased risk of death were (i) low (scores 0–3 vs 4–9) adherence to MD, (ii) obesity [Body Mass Index (BMI) ≥ 30 kg/m^2^ vs <30 kg/m^2^], and (iii) smoking status (current vs. former and current) at recruitment. Age (in years) and education (four levels; categorically modeled) were included as possible confounders. Participants with missing values in any of the above variables were excluded, leaving 15,903 women. Descriptive statistics of all variables included in the analysis are presented in Table 1. In the Cox model, we included three terms for each risk factor, three terms for the two-way product terms between those factors and one for the three-way product term of all three factors.

**Table 1.** Descriptive statistics of the characteristics of 15,903 women participating in analysis.

Continuous variables	
	Mean (sd)
Age (in years), mean (sd)	53.4 (12.5)
Categorical variables	
	*N* (%)
BMI	
Obese (BMI≥30 kg/m^2^)	6206 (39)
Non-obese (BMI<30 kg/m^2^)	9697 (61)
Mediterranean diet	
Low adherence (0–3)	5466 (34%)
Medium-high adherence (4–9)	10,437 (66)
Smoking status	
Current smokers, *n* (%)	3038 (19)
Never-former smokers, *n* (%)	12,865 (81%)
Education	
1st level: no education (<6 years of schooling), *n* (%)	4100 (26)
2nd level: elementary/high school (6–11 years of schooling), *n* (%)	6502 (41)
3rd level: lyceum/technical lyceum (12 years of schooling), *n* (%)	2872 (18)
4th level: at least university degree (>12 years of schooling), *n* (%)	2429 (15)
Mortality	
Alive till the end of follow-up	14699 (92)
Dead during follow-up	1204 (8)

The respective TotRERI_3_, RERI_3_ between the indicated risk factors, as well as their components, i.e. all two- and three-way interactions have been estimated using Equations (A.SB.2)–(A.SB.9) (see Appendix, Section B). The Stata code that was used can be found online on github (https://github.com/mkatsoulis82/Multi-wayinteraction/blob/master/Multi-way20interaction.do), as well as in Appendix (Section B). For the estimation of 95% confidence intervals (CIs), we used the delta method. In Table 2, we present the mortality hazard ratios, of low adherence to MD, obesity, and smoking and of their joint effects as estimated by Cox regression [model (A.SB.1) in the Appendix (section B)].

**Table 2.** Estimated hazard ratios of MD, obesity and smoking and of their product terms from the Cox regression (A.SB.1) from the mortality analysis conducted in women from the EPIC-Greece cohort, along with indexes of additive interaction between low adherence to MD, obesity and smoking.

Results from Cox regression ^a^				
Risk factors of interest and their product terms	*b*	se (*b*)	HR	95% CI for HR
Low MD	0.36	0.09	1.43	1.20, 1.71
High BMI	0.29	0.08	1.34	1.14, 1.56
Current smokers	0.41	0.18	1.51	1.05, 2.16
(low MD) * (high BMI)	−0.27	0.12	0.77	0.60, 0.97
(low MD) * (current smokers)	−0.23	0.30	0.79	0.44, 1.44
(high BMI) * (current smokers)	−0.24	0.29	0.79	0.45, 1.40
(low MD) * (high BMI) * (current smokers)	0.92	0.45	2.51	1.04, 6.02
Relative excess risk due to interaction (RERI)				
	RERI	se (RERI)	95% CI for RERI	
RERI_2_ (low MD, high BMI / never or former smokers)	−0.30	0.17	−0.64, 0.03	
RERI_2_ (low MD, current smokers / low BMI)	−0.23	0.49	−1.19, 0.74	
RERI_2_ (high BMI, current smokers / high MD)	−0.25	0.45	−1.13, 0.63	
RERI_2_ (low MD, high BMI / current smokers)	1.11	0.63	−0.12, 2.35	
RERI_2_ (low MD, current smokers / high BMI)	1.31	0.65	0.05, 2.58	
RERI_2_ (high BMI, current smokers / low MD)	1.20	0.62	−0.01, 2.41	
RERI_3_ (low MD, high BMI, current smokers)	1.98	1.01	0.00, 3.96	
TotRERI_3_ (low MD, high BMI, current smokers)	1.20	0.83	−0.43, 2.82	

### 3.1. Simulations

We conducted a small simulation study to demonstrate the accuracy of the proposed measures for additive interaction. The data generating mechanism and the values chosen for the simulations were informed by our worked example (see above).

The details of these simulations (data generating mechanism, different scenarios tested, results, etc.) are presented in the Appendix, Section H. The scripts of these simulations are in github (https://github.com/mkatsoulis82/Multi-wayinteraction/blob/master/simulations.do).

## 4. Results

In the worked example from the EPIC study, we run a Cox regression model (see details in Section B), where the proportionality assumption held. From Table 2, we conclude that the effects of low MD, obesity and smoking status on mortality were super-additive (TotRERI_3_ = 1.20, even not statistically significant), meaning that there was an extra 120% risk due to the joint presence of all risk factors, compared to the situation that each of them would act separately (see Equation (1)). More specifically, the three-way interaction of these factors beyond the two-way interactions was positive (RERI_3_ = 1.98), indicating that there was a ∼200% excess risk which is explicitly due to the three-way interaction. On the other hand, all the RERI_2_s given the absence of the third risk factor are negative, even not statistically significant (first three rows in Table 2 referring to RERIs), which is an indication that the relative risk from joint action of any two of the following: having low MD score, being a smoker and being obese, when the third factor is absent, is lower compared to sum of the relative risks of these risk factors, when acting separately (sub-additive effects). This means that the excess 120% risk due to the joint presence of all risk factors (TotRERI_3_ = 1.20) is largely due to the three-way interaction of the three risk factors itself (RERI_3_ = 1.98), as the contribution of the two-way interactions is negative (see Equation (2)). Moreover, the corresponding two-way interactions are positive, when the third risk factor is present, which is reflected by the three-way interaction that can be expressed in terms of the two-way interactions [see Equation (4)]. Finally, there was no qualitative interaction in this example.

Moreover, the results from the simulations show negligible biases and CI coverage close to nominal levels (95%) across all the measures of additive interaction. We present the findings of these simulations in detail in the Appendix (Section H).

## 5. Discussion

In this paper, we pointed out the questions of interest in the study of the joint action of >2 binary factors on a health outcome and we proposed the appropriate solutions, by introducing formulae for additive interaction. Previous publications on interactions on the additive scale refer almost exclusively to two-way interactions probably for reasons related to easiness in interpretability and communication of results.

Given this gap in the relevant studies of multi-way interactions on the additive scale, our results are novel for epidemiological research that focuses on the joint action of >2 exposures. We introduced the term ‘total relative excess risk due to interaction (TotRERI)’, a quantity that encompasses all intermediate levels of interaction in the presence of three or more factors. Our formulae enable better interpretability of any evidence for deviations from additivity owned to more than two binary risk factors and provide simple ways of communicating such results from a public health perspective by attributing any excess relative risk to specific combinations of these factors. We would like to mention that these formulae of multi-way interaction on the additive scale cannot be used for ordinal or continuous variables. The corresponding measures of additive interaction are much more complex and difficult to interpret, even in the case of two continuous variables, as we have presented in our previous work [22]; thus the study of multi-way interaction on the additive scale among continuous risk factors was beyond the scope of this paper. Of note, measures of additive interaction can be derived either from models (e.g. logistic regression or Cox regression), as we showed in the worked example, or from contingency tables (see Appendix, Section G). It is very important to mention that all potential interaction terms should be included in the analyses, so that all potential relative risks (by cross-classified exposure status) can be calculated irrespective of whether these interactions are non-significant and could be removed in penalty-based methods. (e.g. Lasso). Otherwise the measures of additive interaction cannot be computed.

Regarding the limitations, researchers should also be concerned whether all possible categories defined by the absence and the presence of the n risk factors include sufficient number of participants, so that all RR’s from expressions (3), for three risk factors, or (6), for *n* risk factors, can be adequately estimated. For case–control studies our results apply for rare diseases only, taking into consideration certain limitations that have expressed in the relevant literature, when using logistic regression [12]. Finally, the problem of the limited power in calculating two-way interactions [17] is also present in multi-way interaction. As observed from our worked example, all the additive interactions were had very wide CIs.

## 6. Conclusions

Given the increasing interest in investigating and evaluating interactions, our results are important for studying multi-way interactions between risk factors and identifying combinations the joint presence of which may be especially important to avoid from a public health perspective.

## Declarations

### Ethics approval and consent to participate

The procedures implemented in the EPIC study were in accordance with the Declaration of Helsinki on the Ethical Principles for Medical Research involving Human Subjects of 1975 as revised in 1983. All study participants signed an informed consent form and the study protocol was approved by the ethics committees of the International Agency for Research on Cancer and the Medical School of the University of Athens.

### Consent for publication

Antonia Trichopoulou is the Principal Investigator for the Greek segment of the EPIC cohort (http://epic.iarc.fr/centers/greece.php). The authors had Antonia Trichopoulou’s permission to use data from the Greek EPIC study (worked example).

## Supplementary Material

Supplemental Material

## Data Availability

The data that has been used is confidential. Nevertheless, the authors provided the scripts to implement multi-way interaction analysis (uploaded on github and presented in the Appendix) and provide recommendations in the paper so that the implementation of multi-way interaction analysis could be straightforward by other researchers.
