# Supplementary material for: Moving from two- to multi-way interactions among binary risk factors on the additive scale
Source: Biostat Epidemiol. 2020 Dec 3;4(1):282–93. doi: 10.1080/24709360.2020.1850171 (PMC8098792; doi:10.1080/24709360.2020.1850171)
Supplement: Supplemental Material [file TBEP_A_1850171_SM9180.pdf]

APPENDIX

Section A – Interaction between 3 risk factors

We have that

$$\mathrm{RERI}_2(X_1, X_2 \mid X_3 = 0) = RR_{X_1+X_2+X_3-} - RR_{X_1+X_2-X_3-} - RR_{X_1-X_2+X_3-} + RR_{X_1-X_2-X_3-} \quad \text{(A.SA.1)}$$

$$\mathrm{RERI}_2(X_1, X_3 \mid X_2 = 0) = RR_{X_1+X_2-X_3+} - RR_{X_1+X_2-X_3-} - RR_{X_1-X_2-X_3+} + RR_{X_1-X_2-X_3-} \quad \text{(A.SA.2)}$$

$$\mathrm{RERI}_2(X_2, X_3 \mid X_1 = 0) = RR_{X_1-X_2+X_3+} - RR_{X_1-X_2+X_3-} - RR_{X_1-X_2-X_3+} + RR_{X_1-X_2-X_3-} \quad \text{(A.SA.3)}$$

So, by replacing in equation 2 (in the paper) to $\mathrm{TotRERI}_3(X_1, X_2, X_3)$,$\mathrm{RERI}_2(X_1, X_2 \mid X_3 = 0)$, $\mathrm{RERI}_2(X_1, X_3 \mid X_2 = 0)$ and $\mathrm{RERI}_2(X_2, X_3 \mid X_1 = 0)$ from equations (1) (in the paper), (A.SA.1), (A.SA.2), (A.SA.3) respectively, then $\mathrm{RERI}_3(X_1, X_2, X_3)$ can be also written as a function of RRs as follows:

$$\begin{aligned}\mathrm{RERI}_3(X_1, X_2, X_3) = {} & RR_{X_1+X_2+X_3+} \\ & -RR_{X_1+X_2+X_3-} - RR_{X_1+X_2-X_3+} - RR_{X_1-X_2+X_3+} \\ & +RR_{X_1+X_2-X_3-} + RR_{X_1-X_2+X_3-} + RR_{X_1-X_2-X_3+} \\ & -RR_{X_1-X_2-X_3-}\end{aligned}$$

as presented in expression 3 (in the paper)

Moreover, we have that

$$\mathrm{RERI}_2(X_1, X_2 \mid X_3 = 1) = \frac{(RR_{X_1+X_2+X_3+} - RR_{X_1+X_2-X_3+} - RR_{X_1-X_2+X_3+} + RR_{X_1-X_2-X_3+})}{RR_{X_1-X_2-X_3+}} \quad \text{(A.SA.4)}$$

$$RERI_2(X_1, X_3|X_2 = 1) = \frac{(RR_{X_1+X_2+X_3+} - RR_{X_1+X_2+X_3-} - RR_{X_1-X_2+X_3+} + RR_{X_1-X_2+X_3-})}{RR_{X_1-X_2+X_3-}} \qquad \text{(A.SA.5)}$$

$$RERI_2(X_2, X_3|X_1 = 1) = \frac{(RR_{X_1+X_2+X_3+} - RR_{X_1+X_2+X_3-} - RR_{X_1+X_2-X_3+} + RR_{X_1+X_2-X_3-})}{RR_{X_1+X_2-X_3-}} \qquad \text{(A.SA.6)}$$

Of note, we standardize $RERI_2$ when the 3rd variable is present by the RR of the 3rd variable, because in the analysis we conduct, all RR's are calculated taking into consideration that $RR_{X_1-X_2-X_3-}$ is the reference relative risk. However, in (A.SA.4), we are interested in

- $RR_{X_1+X_2+}$, given that X_3 is present, compared to $RR_{X_1-X_2-}$, given that X_3 is present, so the relative risk of interest will be $RR_{X_1+X_2+X_3+} / RR_{X_1-X_2-X_3+}$
- $RR_{X_1+X_2-}$, given that X_3 is present, compared to $RR_{X_1-X_2-}$, given that X_3 is present, so the relative risk of interest will be $RR_{X_1+X_2-X_3+} / RR_{X_1-X_2-X_3+}$
- $RR_{X_1-X_2+}$, given that X_3 is present, compared to $RR_{X_1-X_2-}$, given that X_3 is present, so the relative risk of interest will be $RR_{X_1-X_2+X_3+} / RR_{X_1-X_2-X_3+}$
- $RR_{X_1-X_2-}$, given that X_3 is present, compared to $RR_{X_1-X_2-}$, given that X_3 is present, so the relative risk of interest will be $RR_{X_1-X_2-X_3+} / RR_{X_1-X_2-X_3+}$

By combining these bullets, it is straightforward why we standardize by $RR_{X_1-X_2-X_3+}$ in (A.SA.4). For the same reasons we standardized by $RR_{X_1-X_2+X_3-}$ and $RR_{X_1+X_2-X_3-}$ in (A.SA.5) and (A.SA.6).

Moreover, it is also straightforward that if we combine (A.SA.1) and (A.SA.4), or (A.SA.2) and (A.SA.5), or (A.SA.3) and (A.SA.6), we end up to equation (4) in the manuscript.

Section B – Estimation of interactions between 3 risk factors when using Cox (or logistic) regression and implementation in STATA

Estimations using Cox regression

We consider dichotomous variables X_1, X_2 and X_3 as risk factors for the outcome of interest (disease D in incidence analysis or death in mortality analysis) with X_i=(0,1) referring to the absence/presence of risk factor X_i hypothesized to be associated with the lowest/highest risk for disease D). Let also U_j, j=1,2,...,n denote additional variables which serve as potential confounders to the association of X_i with D. Finally, let the hazard rate for disease D at time t be $\lambda(t) = \lambda\left(t, X_1, X_2, X_3, Z_j\right)$ as estimated from Cox regression model including X_1, X_2 and X_3. and additional covariates X_1X_2,X_1X_3, X_2X_3 and $X_1X_2X_3$ for all 2 and 3 way interactions of X_1, X_2 and X_3, and controlling for n covariates U_j, j=1,2,...,n :

$$\lambda(t) = \lambda_0(t) * \exp(a_1X_1 + a_2X_2 + a_3X_3 + a_4X_1X_2 + a_5X_1X_3 + a_6X_2X_3 + a_7X_1X_2X_3 + \textstyle\sum_{j=1}^{n} c_jU_j)\,, \quad \text{(A.SB.1)}$$

where a_k, k=1,..,7 and c_j, j=1,..,N are the log of the hazard ratios estimated from the Cox model. Based on model (A.SB.1), $\text{TotRERI}_3(X_1,X_2,X_3)$ and RERI_3 (X_1, X_2, X_3) can be estimated as shown below:

$$\text{TotRERI}_3(X_1, X_2, X_3) = RR_{X_1+X_2+X_3+} - RR_{X_1+X_2-X_3-} - RR_{X_1-X_2+X_3-} - RR_{X_1-X_2-X_3+} + 2$$

$$= \exp(a_1 + a_2 + a_3 + a_4 + a_5 + a_6 + a_7) - \exp(a_1) - \exp(a_2) - \exp(a_3) + 2 \qquad \text{(A.SB.2)}$$

and

$$\text{RERI}_3(X_1, X_2, X_3) = RR_{X_1+X_2+X_3+} - RR_{X_1+X_2+X_3-} - RR_{X_1+X_2-X_3+} - RR_{X_1-X_2+X_3+}$$

$$+RR_{X_1+X_2-X_3-} + RR_{X_1-X_2+X_3-} + RR_{X_1-X_2-X_3+} - RR_{X_1-X_2-X_3-}$$

$$= \exp(a_1 + a_2 + a_3 + a_4 + a_5 + a_6 + a_7) - \exp(a_1 + a_2 + a_4) - \exp(a_1 + a_3 + a_5)$$

$$-\exp(a_2 + a_3 + a_6) + \exp(a_1) + \exp(a_2) + \exp(a_3) - 1 \quad \text{(A.SB.3)}$$

Similarly, one can estimate also the components of $\text{TotRERI}_3(X_1,X_2,X_3)$ and $\text{RERI}_3(X_1, X_2, X_3)$, based on (A.SB.1):

$$\text{RERI}_2(X_1, X_2|X_3 = 0) = \exp(a_1 + a_2 + a_4) - \exp(a_1) - \exp(a_2) + 1 \quad \text{(A.SB.4)}$$

$$\text{RERI}_2(X_1, X_3|X_2 = 0) = \exp(a_1 + a_3 + a_5) - \exp(a_1) - \exp(a_3) + 1 \quad \text{(A.SB.5)}$$

$$\text{RERI}_2(X_2, X_3|X_1 = 0) = \exp(a_2 + a_3 + a_6) - \exp(a_2) - \exp(a_3) + 1 \quad \text{(A.SB.6)}$$

$$\text{RERI}_2(X_1, X_2|X_3 = 1) = \frac{\exp(a_1+a_2+a_3+a_4+a_5+a_6+a_7) - \exp(a_1+a_3+a_5) - \exp(a_2+a_3+a_6) + \exp(a_3)}{\exp(a_3)} \quad \text{(A.SB.7)}$$

$$\text{RERI}_2(X_1, X_3|X_2 = 1) = \frac{\exp(a_1+a_2+a_3+a_4+a_5+a_6+a_7) - \exp(a_1+a_2+a_4) - \exp(a_2+a_3+a_6) + \exp(a_2)}{\exp(a_2)} \quad \text{(A.SB.8)}$$

$$\text{RERI}_2(X_2, X_3|X_1 = 1) = \frac{\exp(a_1+a_2+a_3+a_4+a_5+a_6+a_7) - \exp(a_1+a_2+a_4) - \exp(a_1+a_3+a_5) + \exp(a_1)}{\exp(a_1)} \quad \text{(A.SB.9)}$$

Implementation in STATA

/*

DESCRIPTION

time; survival time

death (outcome); 0-->alive, 1-->dead

x1 (non adherence to Mediterranean diet - 1st risk factor);

0--> high adherence to Mediterranean diet, 1--> low adherence to Mediterranean diet

x2 (being obese - 2nd risk factor);

0--> not being obese (BMI<30), 1--> being obese (BMI>=30)

x3 (smoking status - 3rd risk factor);

0--> never/former smoker, 1--> current smoker

u1 (age - 1st confounder); continuous in years

u2 (education level - 2nd confounder); categorical in 4 levels

*/

```
* IMPLEMENTATION
* At first we compute the product terms
gen x1x2=x1*x2
gen x1x3=x1*x3
gen x2x3=x2*x3
gen x1x2x3=x1*x2*x3

*We run the Cox model
stset time, failure(death)

stcox x1 x2 x3 x1x2 x1x3 x2x3 x1x2x3 u1 i.u2

*We compute TotRERI3
nlcom TotRERI3: exp(_b[x1]+_b[x2]+_b[x3]+_b[x1x2]+_b[x1x3]+_b[x2x3]+_b[x1x2x3])-
exp(_b[x1])-exp(_b[x2])-exp(_b[x3])+2

*We compute RERI3
nlcom RERI3:    exp(_b[x1]+_b[x2]+_b[x3]+_b[x1x2]+_b[x1x3]+_b[x2x3]+_b[x1x2x3])-
exp(_b[x1]+_b[x2]+_b[x1x2])-exp(_b[x1]+_b[x3]+_b[x1x3])-
exp(_b[x2]+_b[x3]+_b[x2x3])+exp(_b[x1])+exp(_b[x2])+exp(_b[x3])-1
```

*We compute 2-way interactions, given the 3rd risk factor is absent

*RERI(x1,x2/x3=0)

nlcom RERI2_x1_x2_given_x3is0: exp(_b[x1]+_b[x2]+_b[x1x2])-exp(_b[x1])-exp(_b[x2])+1

*RERI(x1,x3/x2=0)

nlcom RERI2_x1_x3_given_x2is0: exp(_b[x1]+_b[x3]+_b[x1x3])-exp(_b[x1])-exp(_b[x3])+1

*RERI(x2,x3/x1=0)

nlcom RERI2_x2_x3_given_x1is0: exp(_b[x2]+_b[x3]+_b[x2x3])-exp(_b[x2])-exp(_b[x3])+1

*We compute 2-way interactions, given the 3rd risk factor is present

*RERI(x1,x2/x3=1)

nlcom RERI2_x1_x2_given_x3is1:
(exp(_b[x1]+_b[x2]+_b[x3]+_b[x1x2]+_b[x1x3]+_b[x2x3]+_b[x1x2x3])-
exp(_b[x1]+_b[x3]+_b[x1x3])-exp(_b[x2]+_b[x3]+_b[x2x3])+exp(_b[x3]))/exp(_b[x3])

*RERI(x1,x3/x2=1)

nlcom RERI2_x1_x3_given_x2is1:
(exp(_b[x1]+_b[x2]+_b[x3]+_b[x1x2]+_b[x1x3]+_b[x2x3]+_b[x1x2x3])-
exp(_b[x1]+_b[x2]+_b[x1x2])-exp(_b[x2]+_b[x3]+_b[x2x3])+exp(_b[x2]))/exp(_b[x2])

*RERI(x2,x3/x1=1)

nlcom RERI2_x2_x3_given_x1is1:
(exp(_b[x1]+_b[x2]+_b[x3]+_b[x1x2]+_b[x1x3]+_b[x2x3]+_b[x1x2x3])-
exp(_b[x1]+_b[x2]+_b[x1x2])-exp(_b[x1]+_b[x3]+_b[x1x3])+exp(_b[x1]))/exp(_b[x1])

*The same formulae for all these RERIs are used when running logistic regression

* CHECK FOR QUALITATIVE INTERACTION

*We run again the Cox model

stset time, failure(death)

stcox x1 x2 x3 x1x2 x1x3 x2x3 x1x2x3 u1 i.u2

* To check whether the risk for x1 is increasing across strata of x2,x3, we have to examine whether the following quantities are positive (i.e. >0)

* 1a) to see if RR100>RR000, we check whether RR100-RR000>0

disp exp(_b[x1])-1

* 1b) to see if RR110>RR010, we check whether RR110-RR010>0

disp exp(_b[x1]+_b[x2]+_b[x1x2])-exp(_b[x2])

* 1c) to see if RR101>RR001, we check whether RR101-RR001>0

disp exp(_b[x1]+_b[x3]+_b[x1x3])-exp(_b[x3])

* 1d) to see if RR111>RR011, we check whether RR111-RR011>0

disp exp(_b[x1]+_b[x2]+_b[x3]+_b[x1x2]+_b[x1x3]+_b[x2x3]+_b[x1x2x3])-exp(_b[x2]+_b[x3]+_b[x2x3])

* To check whether the risk for x2 is increasing across strata of x1,x3

* 2a) to see if RR010>RR000, we check whether RR010-RR000>0

disp exp(_b[x2])-1

* 2b) to see if RR110>RR100, we check whether RR110-RR100>0

disp exp(_b[x1]+_b[x2]+_b[x1x2])-exp(_b[x1])

* 2c) to see if RR011>RR001, we check whether RR011-RR001>0

disp exp(_b[x2]+_b[x3]+_b[x2x3])-exp(_b[x3])

* 2d) to see if RR111>RR101, we check whether RR111-RR101>0

disp exp(_b[x1]+_b[x2]+_b[x3]+_b[x1x2]+_b[x1x3]+_b[x2x3]+_b[x1x2x3])-exp(_b[x1]+_b[x3]+_b[x1x3])

* To check whether the risk for x3 is increasing across strata of x1,x2

* 2a) to see if RR001>RR000, we check whether RR001-RR000>0

disp exp(_b[x3])-1

* 2b) to see if RR101>RR100, we check whether RR101-RR010>0

disp exp(_b[x1]+_b[x3]+_b[x1x3])-exp(_b[x1])

* 2c) to see if RR011>RR010, we check whether RR011-RR100>0

disp exp(_b[x2]+_b[x3]+_b[x2x3])-exp(_b[x2])

* 2d) to see if RR111>RR110, we check whether RR111-RR110>0

disp exp(_b[x1]+_b[x2]+_b[x3]+_b[x1x2]+_b[x1x3]+_b[x2x3]+_b[x1x2x3])-exp(_b[x1]+_b[x2]+_b[x1x2])

Section C – proofs of equations of multi way interaction

Proof of expression (6)

We define the excess relative risk by $ERR_{X_1\#X_2\#\ldots X_n\#}$, where # = +/-, as

$$ERR_{X_1\#X_2\#\ldots X_n\#} = RR_{X_1\#X_2\#\ldots X_n\#} - RR_{X_1-X_2-\cdots X_n-}$$, i.e.

$$ERR_{X_1\#X_2\#\ldots X_n\#} = RR_{X_1\#X_2\#\ldots X_n\#} - 1$$

Now, expression (5) from the text can be written as

$$TotRERI_n(X_1, X_2, \ldots, X_n) = ERR_{X_1+X_2+\cdots X_n+}$$

$$-ERR_{X_1+X_2-\cdots X_n-} - ERR_{X_1-X_2+\cdots X_n-} - \cdots - ERR_{X_1-X_2-\cdots X_n+} \qquad \text{(A.SC.1)}$$

$TotRERI_n$ can be expressed in terms of relative risks (see equation 5 in the paper), but also as a function of all interactions, more specifically all $RERI_k$ for k≤n, that is

$$TotRERI_n(X_1, X_2, \ldots, X_n) = RERI_n(X_1, X_2, \ldots, X_n)$$

$$+ \sum_{\binom{n}{n-1}} RERI_{n-1}(X_1, X_2, \ldots, X_n | 1 \text{ of the } X_i = 0)$$

$$+ \sum_{\binom{n}{n-2}} RERI_{n-2}(X_1, X_2, \ldots, X_n | 2 \text{ of the } X_i = 0)$$

$$\dots$$

$$+\sum_{\binom{n}{2}} \mathrm{RERI}_2(X_1, X_2, \dots, X_n | (n-2) \text{ of the } X_i = 0) \qquad (A.SC.2)$$

Now, to calculate RERI_n, we can combine equations (A.SC.1) and (A.SC.2), we have to solve the recurrence relation

$$\mathrm{RERI}_n + \sum_{\binom{n}{n-1}} \mathrm{RERI}_{n-1} + \sum_{\binom{n}{n-2}} \mathrm{RERI}_{n-2} + \cdots + \sum_{\binom{n}{2}} \mathrm{RERI}_2 = \mathrm{ERR}_{X_1+X_2+\cdots X_n+} - \mathrm{ERR}_{X_1+X_2-\cdots X_n-} - \mathrm{ERR}_{X_1-X_2+\cdots X_n-} - \cdots - \mathrm{ERR}_{X_1-X_2-\cdots X_n+}$$

Where $\mathrm{RERI}_{n-k} = \mathrm{RERI}_k(X_1, X_2, \dots, X_n | (n-k) \text{ of the } X_i = 0)$

In other words, we have to solve

$$\mathrm{RERI}_n = \mathrm{ERR}_{X_1+X_2+\cdots X_n+} - \mathrm{ERR}_{X_1+X_2-\cdots X_n-} - \mathrm{ERR}_{X_1-X_2+\cdots X_n-} - \cdots - \mathrm{ERR}_{X_1-X_2-\cdots X_n+} - \sum_{\binom{n}{1}} \mathrm{RERI}_{n-1} - \sum_{\binom{n}{2}} \mathrm{RERI}_{n-2} - \cdots - \sum_{\binom{n}{n-2}} \mathrm{RERI}_2$$

using as notation $\mathrm{RR}_{(k)} = \mathrm{RR}_{k \text{ of the n } X_i's=1, \text{the rest } (n-k)\ X_i's=0}$ we have that

$$\mathrm{RERI}_n = \mathrm{RR}_{(n)} - \mathrm{RR}_{(0)} - \sum_{\binom{n}{1}} \mathrm{ERR}_{(1)} - \sum_{\binom{n}{n-1}} \mathrm{RERI}_{n-1} - \sum_{\binom{n}{n-2}} \mathrm{RERI}_{n-2} - \cdots - \sum_{\binom{n}{2}} \mathrm{RERI}_2$$

in other words

$$\mathrm{RERI}_n = \mathrm{RR}_{(n)} - \sum_{\binom{n}{n-1}} \mathrm{RERI}_{n-1} - \sum_{\binom{n}{n-2}} \mathrm{RERI}_{n-2} - \cdots - \sum_{\binom{n}{2}} \mathrm{RERI}_2 - \sum_{\binom{n}{1}} \mathrm{ERR}_{(1)} - \sum_{\binom{n}{0}} \mathrm{RR}_{(0)} \qquad (A.SC.3)$$

when we express all the (different) RERI_{n-1}'s from the recurrence relation (A.SC.3), we have that

$$
\begin{aligned}
\mathrm{RERI_n} = {} & \mathrm{RR_{(n)}} - \sum_{\binom{n}{n-1}} \mathrm{RR_{(n-1)}} + \sum_{\binom{n}{n-1}} \sum_{\binom{n-1}{n-2}} \mathrm{RERI_{n-2}} + \sum_{\binom{n}{n-1}} \sum_{\binom{n-1}{n-3}} \mathrm{RERI_{n-3}} + \cdots + \sum_{\binom{n}{n-1}} \sum_{\binom{n-1}{n-k}} \mathrm{RERI_{n-k}} + \cdots + \sum_{\binom{n}{n-1}} \sum_{\binom{n-1}{1}} \mathrm{ERR_1} + \sum_{\binom{n}{n-1}} \sum_{\binom{n-1}{0}} \mathrm{RR_0} \\
& - \sum_{\binom{n}{n-2}} \mathrm{RERI_{n-2}} - \sum_{\binom{n}{n-3}} \mathrm{RERI_{n-3}} - \ldots - \sum_{\binom{n}{k}} \mathrm{RERI_{n-k}} - \ldots - \sum_{\binom{n}{1}} \mathrm{ERR_1} - \sum_{\binom{n}{0}} \mathrm{RR_0} \qquad (\mathrm{A.SC.4})
\end{aligned}
$$

and when we express all $\mathrm{RERI_{n-2}}$’s from (A.SC.4) using the recurrence relation (A.SC.3), we have that

$$
\begin{aligned}
\mathrm{RERI_n} = {} & \mathrm{RR_{(n)}} - \sum_{\binom{n}{n-1}} \mathrm{RR_{(n-1)}} + \left(\sum_{\binom{n}{n-1}} \sum_{\binom{n-1}{n-2}} \mathrm{RR_{(n-2)}} - \sum_{\binom{n}{n-2}} \mathrm{RR_{(n-2)}} \right) \\
& - \sum_{\binom{n}{n-1}} \sum_{\binom{n-1}{n-2}} \sum_{\binom{n-2}{n-3}} \mathrm{RERI_{n-3}} - \sum_{\binom{n}{n-1}} \sum_{\binom{n-1}{n-2}} \sum_{\binom{n-2}{n-4}} \mathrm{RERI_{n-4}} - \cdots - \sum_{\binom{n}{n-1}} \sum_{\binom{n-1}{n-2}} \sum_{\binom{n-2}{n-k}} \mathrm{RERI_{n-k}} - \cdots - \sum_{\binom{n}{n-1}} \sum_{\binom{n-1}{n-2}} \sum_{\binom{n-2}{0}} \mathrm{RR_0} \\
& + \sum_{\binom{n}{n-1}} \sum_{\binom{n-1}{n-3}} \mathrm{RERI_{n-3}} + \sum_{\binom{n}{n-1}} \sum_{\binom{n-1}{n-4}} \mathrm{RERI_{n-4}} + \ldots + \sum_{\binom{n}{n-1}} \sum_{\binom{n-1}{n-k}} \mathrm{RERI_{n-k}} + \ldots + \sum_{\binom{n}{n-1}} \sum_{\binom{n-1}{0}} \mathrm{RR_0} \\
& + \sum_{\binom{n}{n-2}} \sum_{\binom{n-2}{n-3}} \mathrm{RERI_{n-3}} + \sum_{\binom{n}{n-2}} \sum_{\binom{n-2}{n-4}} \mathrm{RERI_{n-4}} + \ldots + \sum_{\binom{n}{n-2}} \sum_{\binom{n-2}{n-k}} \mathrm{RERI_{n-k}} + \ldots + \sum_{\binom{n}{n-2}} \sum_{\binom{n-2}{0}} \mathrm{RR_0} \\
& - \sum_{\binom{n}{n-3}} \mathrm{RERI_{n-3}} - \sum_{\binom{n}{n-4}} \mathrm{RERI_{n-4}} - \ldots - \sum_{\binom{n}{k}} \mathrm{RERI_{n-k}} - \ldots - \sum_{\binom{n}{0}} \mathrm{RR_0} \qquad (\mathrm{A.SC.5})
\end{aligned}
$$

of interest from (A.SC.5) to compute the quantity

$$\sum_{\binom{n}{n-1}} \sum_{\binom{n-1}{n-2}} RR_{(n-2)} - \sum_{\binom{n}{n-2}} RR_{(n-2)} \qquad (A.SC.6)$$

i.e. how many time we are going to sum up $RR_{(n-2)}$

from combinatorics, it is known that

$$\binom{n}{r}\binom{r}{k} = \binom{n}{k}\binom{n-k}{r-k}$$

so from (A.SC.6) we have that

$$\binom{n}{n-1}\binom{n-1}{n-2} = \binom{n}{n-2}\binom{n-n+2}{n-1-n+2} = \binom{n}{n-2}\binom{2}{1} = 2\binom{n}{n-2}$$

in other words, $\binom{n}{n-1}\binom{n-1}{n-2} - \binom{n}{n-2} = 2\binom{n}{n-2} - \binom{n}{n-2} = \binom{n}{n-2}$

so it seems that

$$\sum_{\binom{n}{n-1}} \sum_{\binom{n-1}{n-2}} RR_{(n-2)} - \sum_{\binom{n}{n-2}} RR_{(n-2)} = \sum_{\binom{n}{n-2}} RR_{(n-2)} \qquad (A.SC.7)$$

However, to prove (A.SC.7) and more specifically that the summation of $RR_{(n-2)}$ will be the sum of all different $RR_{(n-2)}$'s, we work as follows;

It is obvious that the part of the summation of $RR_{(n-2)}$, which is created through the route

$RERI_n \rightarrow RERI_{n-2}$ is equal to

$$\sum_{\binom{n}{n-2}} RR_{(n-2)}$$

more specifically it is equal the sum of all different $RR_{(n-2)}$'s

Now, we have to prove that the part of the summation of $RR_{(n-2)}$, which is created through the route

$RERI_n \rightarrow RERI_{n-1} \rightarrow RERI_{n-2}$, which is

$$\sum_{\binom{n}{n-1}} \sum_{\binom{n-1}{n-2}} RR_{(n-2)}$$

can be written

$$\sum_{\binom{n}{n-1}} \sum_{\binom{n-1}{n-2}} RR_{(n-2)} = 2 * \sum_{\binom{n}{n-2}} RR_{(n-2)}$$

In other words, that is equal to the sum of the $\binom{n}{n-2}$ different $RERI_{n-2}$, multiplied by 2

To show that, we work as follows;

We have $\binom{n}{n-1} = n$ different $RERI_{n-1}$'s that lead to $\binom{n}{n-1}\binom{n-1}{n-2} = 2 * \binom{n}{n-2} = 2 * \frac{(n-1)(n-2)}{2}$ $RERI_{n-2}$'s

i.e. $(n-1)(n-2)$ $RERI_{n-2}$'s through the following pattern

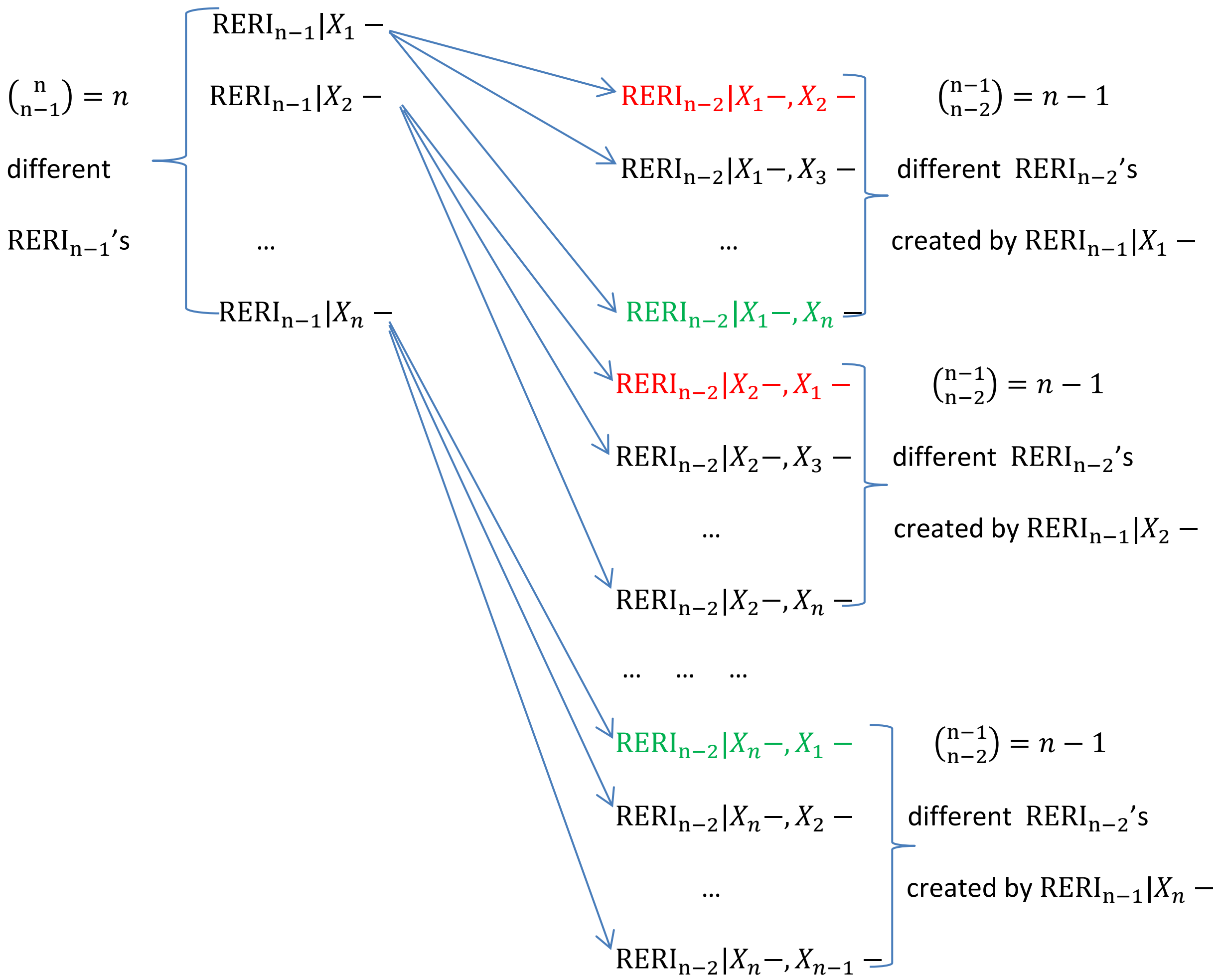

$\binom{n}{n-1} = n$
different
RERI_{n-1}'s
$\mathrm{RERI}_{n-1}|X_1-$
$\mathrm{RERI}_{n-1}|X_2-$
…
$\mathrm{RERI}_{n-1}|X_n-$
$\mathrm{RERI}_{n-2}|X_1-, X_2-$
$\mathrm{RERI}_{n-2}|X_1-, X_3-$
…
$\mathrm{RERI}_{n-2}|X_1-, X_n-$
$\binom{n-1}{n-2} = n-1$
different RERI_{n-2}'s
created by $\mathrm{RERI}_{n-1}|X_1-$
$\mathrm{RERI}_{n-2}|X_2-, X_1-$
$\mathrm{RERI}_{n-2}|X_2-, X_3-$
…
$\mathrm{RERI}_{n-2}|X_2-, X_n-$
$\binom{n-1}{n-2} = n-1$
different RERI_{n-2}'s
created by $\mathrm{RERI}_{n-1}|X_2-$
… … …
$\mathrm{RERI}_{n-2}|X_n-, X_1-$
$\mathrm{RERI}_{n-2}|X_n-, X_2-$
…
$\mathrm{RERI}_{n-2}|X_n-, X_{n-1}-$
$\binom{n-1}{n-2} = n-1$
different RERI_{n-2}'s
created by $\mathrm{RERI}_{n-1}|X_n-$

From above, we can observe that the $\binom{n}{n-2}$ different RERI_{n-2} are created from the $\binom{n}{n-1} = n$ different RERI_{n-1}'s twice. This is done because $\mathrm{RERI}_{n-2}|X_m-, X_l-$ is derived by both $\mathrm{RERI}_{n-1}|X_m-$ and $\mathrm{RERI}_{n-1}|X_l-$, but from no other $\mathrm{RERI}_{n-1}|X_j-$, j≠m, j≠l (for example see $\mathrm{RERI}_{n-2}|X_1-, X_2-$ and $\mathrm{RERI}_{n-2}|X_1-, X_n-$). So the total $\mathrm{RR}_{(n-2)}$ that will be created through the route $\mathrm{RERI}_n \to \mathrm{RERI}_{n-1} \to \mathrm{RERI}_{n-2}$ is equal to

$$\sum_{\binom{n}{n-1}} \sum_{\binom{n-1}{n-2}} \mathrm{RR}_{(n-2)} = 2 * \sum_{\binom{n}{n-2}} \mathrm{RR}_{(n-2)}$$

After this result, (A.SC.7) is proved, because

$$\sum_{\binom{n}{n-1}} \sum_{\binom{n-1}{n-2}} \mathrm{RR}_{(n-2)} - \sum_{\binom{n}{n-2}} \mathrm{RR}_{(n-2)} = 2 * \sum_{\binom{n}{n-2}} \mathrm{RR}_{(n-2)} - \sum_{\binom{n}{n-2}} \mathrm{RR}_{(n-2)} = \sum_{\binom{n}{n-2}} \mathrm{RR}_{(n-2)}$$

Furthermore, when we express RERI_{n-3} from (A.SC.5) using the recurrence relation (A.SC.3), we have that

$$\mathrm{RERI}_n = \mathrm{RR}_{(n)} - \sum_{\binom{n}{n-1}} \mathrm{RR}_{(n-1)} + \sum_{\binom{n}{n-2}} \mathrm{RR}_{(n-2)} - \left(\sum_{\binom{n}{n-1}} \sum_{\binom{n-1}{n-2}} \sum_{\binom{n-2}{n-3}} \mathrm{RR}_{(n-3)} - \sum_{\binom{n}{n-1}} \sum_{\binom{n-1}{n-3}} \mathrm{RR}_{(n-3)} - \sum_{\binom{n}{n-2}} \sum_{\binom{n-2}{n-3}} \mathrm{RR}_{(n-3)} + \sum_{\binom{n}{n-3}} \mathrm{RR}_{(n-3)} \right)$$

$$+ \sum_{\binom{n}{n-1}} \sum_{\binom{n-1}{n-2}} \sum_{\binom{n-2}{n-3}} \sum_{\binom{n-3}{n-4}} \mathrm{RERI}_{n-4} + \sum_{\binom{n}{n-1}} \sum_{\binom{n-1}{n-2}} \sum_{\binom{n-2}{n-3}} \sum_{\binom{n-3}{n-5}} \mathrm{RERI}_{n-5} + \cdots + \sum_{\binom{n}{n-1}} \sum_{\binom{n-1}{n-2}} \sum_{\binom{n-2}{n-3}} \sum_{\binom{n-3}{n-k}} \mathrm{RERI}_{n-k} + \cdots + \sum_{\binom{n}{n-1}} \sum_{\binom{n-1}{n-2}} \sum_{\binom{n-2}{n-3}} \sum_{\binom{n-3}{0}} \mathrm{RR}_0$$

… … … … …

$$\cdots \qquad \cdots \qquad \cdots \qquad \cdots \qquad \cdots$$

$$\cdots \qquad \cdots \qquad \cdots \qquad \cdots \qquad \cdots$$

$$-\sum_{\binom{n}{n-4}} RERI_{n-4} \quad - \quad \sum_{\binom{n}{n-5}} RERI_{n-5} - \quad \ldots \quad -\sum_{\binom{n}{k}} RERI_{n-k} \quad -\sum_{\binom{n}{0}} RR_0 \qquad (A.SC.8)$$

of interest from (A.SC.8) to compute how many time we are going to sum up $RR_{(n-3)}$, i.e.

$$\sum_{\binom{n}{n-1}} \sum_{\binom{n-1}{n-2}} \sum_{\binom{n-2}{n-3}} RR_{(n-3)} - \sum_{\binom{n}{n-1}} \sum_{\binom{n-1}{n-3}} RR_{(n-3)} - \sum_{\binom{n}{n-2}} \sum_{\binom{n-2}{n-3}} RR_{(n-3)} + \sum_{\binom{n}{n-3}} RR_{(n-3)} \qquad (A.SC.9)$$

We have that

$$\binom{n}{n-1}\binom{n-1}{n-2}\binom{n-2}{n-3} = 6\binom{n}{n-3}, \qquad \binom{n}{n-1}\binom{n-1}{n-3} = 3\binom{n}{n-3}, \qquad \binom{n}{n-2}\binom{n-2}{n-3} = 3\binom{n}{n-3}, so$$

$$6\binom{n}{n-3} - 3\binom{n}{n-3} - 3\binom{n}{n-3} + \binom{n}{n-3} = \binom{n}{n-3}$$

so if we work as we did to show (A.SC.7), we will find that

$$\left(\sum_{\binom{n}{n-1}}\sum_{\binom{n-1}{n-2}}\sum_{\binom{n-2}{n-3}} RR_{(n-3)} - \sum_{\binom{n}{n-1}}\sum_{\binom{n-1}{n-3}} RR_{(n-3)} - \sum_{\binom{n}{n-2}}\sum_{\binom{n-2}{n-3}} RR_{(n-3)} + \sum_{\binom{n}{n-3}} RR_{(n-3)}\right) = \sum_{\binom{n}{n-3}} RR_{(n-3)}$$

In other words, (A.SC.9) is equal to the sum of the $\binom{n}{n-3}$ different $RR_{(n-3)}$, that come from the application of (A.SC.3) for the conversion of $\binom{n}{n-3}$ different $RERI_{(n-3)}$'s.

Moreover, for the parenthesis related to the summation of $RR_{(n-3)}$ (i.e. (A.SC.9) we may also calculate it as follows:

We let

$\sum_{\binom{n}{n-1}}\sum_{\binom{n-1}{n-2}}\sum_{\binom{n-2}{n-3}} RR_{(n-3)} \rightarrow (1\ \ 2)_{RR_{(n-3)}}$, i.e. the part of the summation that $RR_{(n-3)}$ is calculated from $RERI_{n-3}$through the route

$RERI_n \rightarrow RERI_{n-1} \rightarrow RERI_{n-2} \rightarrow RERI_{n-3}$ (we name points [1] and [2]),

i.e. we go from $RERI_n$to $RERI_{n-3}$through $RERI_{n-1}$to $RERI_{n-2}$ (points [1] and [2] respectively)

$\sum_{\binom{n}{n-1}}\sum_{\binom{n-1}{n-3}} RR_{(n-3)} \rightarrow (1)_{RR_{(n-3)}}$, i.e. the part of the summation that $RR_{(n-3)}$ is calculated from $RERI_{n-3}$through the route

$RERI_n \rightarrow RERI_{n-1} \rightarrow RERI_{n-3}$ (we name point [1]),

$\sum_{\binom{n}{n-2}}\sum_{\binom{n-2}{n-3}} RR_{(n-3)} \rightarrow (2)_{RR_{(n-3)}}$, i.e. the part of the summation that $RR_{(n-3)}$ is calculated from $RERI_{n-3}$through the route

$RERI_n \rightarrow RERI_{n-2} \rightarrow RERI_{n-3}$ (we name point [2]),

$\sum_{\binom{n}{n-3}} RR_{(n-3)} \rightarrow (\emptyset)_{RR_{(n-3)}}$, i.e. the part of the summation that $RR_{(n-3)}$ is calculated from $RERI_{n-3}$ through the direct route

$RERI_n \rightarrow RERI_{n-3}$ (we name no point [Ø]),

We also name the summation $\{1 \;\; 2\}_{RR_{(n-3)}}$ as follows

$$\{1 \;\; 2\}_{RR_{(n-3)}} = (1 \;\; 2)_{RR_{(n-3)}} - (1)_{RR_{(n-3)}} - (2)_{RR_{(n-3)}} + (\emptyset)_{RR_{(n-3)}} = \sum_{\binom{n}{n-1}} \sum_{\binom{n-1}{n-2}} \sum_{\binom{n-2}{n-3}} RR_{(n-3)} - \sum_{\binom{n}{n-1}} \sum_{\binom{n-1}{n-3}} RR_{(n-3)} - \sum_{\binom{n}{n-2}} \sum_{\binom{n-2}{n-3}} RR_{(n-3)} + \sum_{\binom{n}{n-3}} RR_{(n-3)}$$

On the same way we have that $\{1\}_{RR_{(n-2)}} = (1)_{RR_{(n-2)}} - (\emptyset)_{RR_{(n-2)}}$, because $\{1\}_{RR_{(n-2)}} = \sum_{\binom{n}{n-1}} \sum_{\binom{n-1}{n-2}} RR_{(n-2)} - \sum_{\binom{n}{n-2}} RR_{(n-2)}$

All routes of $\{1\}_{RR_{(n-2)}}$ are multiples of $\binom{n}{n-2}$, i.e. because of (A.SC.7) we have

$(1)_{RR_{(n-2)}} = \text{sum of} \binom{n}{n-2}$ *different* $RR_{(n-2)}$, multiplied by b_1, where $b_1 = 2$

$(\emptyset)_{RR_{(n-2)}} = \text{sum of} \binom{n}{n-2}$ *different* $RR_{(n-2)}$, multiplied by b_2, where $b_2 = 1$

And on the same way, for $\{1 \;\; 2\}_{RR_{(n-3)}}$, all routes of $\{1 \;\; 2\}_{RR_{(n-3)}}$ are multiples of $\binom{n}{n-3}$

$(1 \;\; 2)_{RR_{(n-3)}} = \text{sum of} \binom{n}{n-3}$ *different* $RR_{(n-3)}$, multiplied by c_1, where $c_1 = 6$

$(1)_{RR_{(n-3)}} = \text{sum of} \binom{n}{n-3}$ *different* $RR_{(n-3)}$, multiplied by c_2, where $c_2 = 3$

$(2)_{RR_{(n-3)}} = \text{sum of} \binom{n}{n-3}\ \textit{different}\ RR_{(n-3)}$, multiplied by c_3, where $c_3 = 3$

$(Ø)_{RR_{(n-3)}} = \text{sum of} \binom{n}{n-3}\ \textit{different}\ RR_{(n-3)}$, multiplied by c_4, where $c_4 = 1$

We further observe that the summation $\{1\ \ 2\}_{RR_{(n-3)}}$ has q=2 points and $2^q=2^2=4$ routes and are constructed as follows

a) half of the $\{1\ \ 2\}_{RR_{(n-3)}}$ routes ($=2^{2-1}$ =2 routes) have point [2] as last point. This means that

half of the $\{1\ \ 2\}_{RR_{(n-3)}}$ routes ($=2^{2-1}$ =2 routes) are made by the $\{1\}_{RR_{(n-2)}}$ route after adding point [2] by summing up all $\sum_{\binom{n-2}{n-3}} RR_{(n-3)}$, i.e.

$$(1\ \ 2)_{RR_{(n-3)}}, \text{i.e.}\ RERI_n \to RERI_{n-1} \to RERI_{n-2} \to RERI_{n-3} \rightarrow \sum_{\binom{n}{n-1}} \sum_{\binom{n-1}{n-2}} \sum_{\binom{n-2}{n-3}} RR_{(n-3)} \qquad \text{(A.SC.10)}$$

and $$(2)_{RR_{(n-3)}} \quad \text{i.e.} = RERI_n \to RERI_{n-2} \to RERI_{n-3} \rightarrow \sum_{\binom{n}{n-2}} \sum_{\binom{n-2}{n-3}} RR_{(n-3)} \qquad \text{(A.SC.11)}$$

We name $\{1\ \ 2\}_{RR_{(n-3);[i]}}$ the routes of $\{1\ \ 2\}_{RR_{(n-3)}}$ having as last point [i], where i=0,1 or 2.

In other words, we name $\{1\ \ 2\}_{RR_{(n-3);[i]}}$ all the routes $RERI_n \to \cdots \to RERI_{n-i} \to RERI_{n-3}$

For i=2 we find that

since $\{1\}_{RR_{(n-2)}} = \sum_{\binom{n}{n-2}} RR_{(n-2)}$, and we have that $\{1\ \ 2\}_{RR_{(n-3);[2]}} = \sum_{\binom{n}{n-2}} \sum_{\binom{n-2}{n-3}} RR_{(n-3)} = \binom{3}{1} * \sum_{\binom{n}{n-3}} RR_{(n-3)}$

$$\{1 \quad 2\}_{RR_{(n-3);[2]}} = \sum_{\binom{n}{n-1}} \sum_{\binom{n-1}{n-2}} \sum_{\binom{n-2}{n-3}} RR_{(n-3)} - \sum_{\binom{n}{n-2}} \sum_{\binom{n-2}{n-3}} RR_{(n-3)} = \sum_{\binom{n}{n-1}} \sum_{\binom{n-1}{n-2}} \sum_{\binom{n-2}{n-3}} RR_{(n-3)} - \sum_{\binom{n}{n-2}} \sum_{\binom{n-2}{n-3}} RR_{(n-3)} =$$

But due to (A.SC.7), we have that

$$= 2 * \sum_{\binom{n}{n-2}} \sum_{\binom{n-2}{n-3}} RR_{(n-3)} - \sum_{\binom{n}{n-2}} \sum_{\binom{n-2}{n-3}} RR_{(n-3)} = \sum_{\binom{n}{n-2}} \sum_{\binom{n-2}{n-3}} RR_{(n-3)}$$

b) 2^{2-2} =1 route of $\{1 \quad 2\}_{RR_{(n-3)}}$ is constructed having as last point [1], i.e. $\{1 \quad 2\}_{RR_{(n-3);[1]}}$

$$\{1 \quad 2\}_{RR_{(n-3);[1]}} = (1)_{RR_{(n-3)}}, \text{i.e.} \, RERI_n \to RERI_{n-1} \to RERI_{n-3} \rightarrow \sum_{\binom{n}{n-1}} \sum_{\binom{n-1}{n-3}} RR_{(n-3)} \qquad \text{(A.SC.12)}$$

Since $\{\emptyset\}_{RR_{(n-1)}} = \sum_{\binom{n}{n-1}} RR_{(n-1)}$, we have that $\{1 \quad 2\}_{RR_{(n-3);[1]}} = \sum_{\binom{n}{n-1}} \sum_{\binom{n-1}{n-3}} RR_{(n-3)} = \binom{3}{1} * \sum_{\binom{n}{n-3}} RR_{(n-3)}$

c) 1 more route of $\{1 \quad 2\}_{RR_{(n-3)}}$ is constructed having as last point [0], i.e. $\{1 \quad 2\}_{RR_{(n-3);[1]}}$

$$\{1 \quad 2\}_{RR_{(n-3);[0]}} = (\emptyset)_{RR_{(n-3)}} \, \text{i.e.} \, RERI_n \to RERI_{n-1} \to RERI_{n-3} \rightarrow \sum_{\binom{n}{n-3}} RR_{(n-3)}, \qquad \text{(A.SC.13)}$$

So we have that

$$\{1 \quad 2\}_{RR_{(n-3)}} = \sum_{j=1}^{3} (-1)^{(j-1)} \sum_{\binom{n}{n-(3-j)}} \sum_{\binom{n-(3-j)}{n-3}} RR_{(n-3)} \qquad \text{(A.SC. 14)}$$

Now, we are ready to solve (A.SC.3), by applying complete induction to show that the summation of all $\mathrm{RR}_{(n-k)}$, for all k=1,2,...n when we replace all $\mathrm{RERI}_{(n)}$ up to $\mathrm{RERI}_{(n-k)}$ in (A.SC.3) is $\sum_{\binom{n}{n-k}} \mathrm{RR}_{(n-k)}$

In order to prove that, we apply step by step complete induction

1) for i=1, the summation for $\mathrm{RR}_{(n-1)}$ in (A.SC.3) is (see (A.SC.4))

$$\sum_{\binom{n}{n-1}} \mathrm{RR}_{(n-1)}$$

i.e. our hypothesis hold

2) for all i=2,3,...k-1, the summation for $\mathrm{RR}_{(n-i)}$ in (A.SC.3) is

$$\sum_{\binom{n}{n-i}} \mathrm{RR}_{(n-i)}$$

3) for i=k, we have to show that the summation for $\mathrm{RR}_{(n-k)}$ when we replace all $\mathrm{RERI}_{(n)}$ up to $\mathrm{RERI}_{(n-k)}$ in (A.SC.3) is

$$\sum_{\binom{n}{n-k}} \mathrm{RR}_{(n-k)} \qquad \text{(A.SC.15)}$$

To prove (A.SC.15), we have to show that

$$\{1 \quad 2 \quad 3 \quad \ldots \quad (k-2) \quad (k-1)\}_{\mathrm{RR}_{(n-k);[i]}} = \sum_{\binom{n}{n-i}} \sum_{\binom{n-i}{n-k}} \mathrm{RR}_{(n-k)} = \binom{k}{i} * \sum_{\binom{n}{n-k}} \mathrm{RR}_{(n-k)} \qquad \text{(A.SC.16)}$$

In other words, from (A.SC.16), we have all the different $\mathrm{RR}_{(\mathrm{n-k})}$ appear $\binom{\mathrm{k}}{\mathrm{i}}$ times from the routes of

$\{1 \quad 2 \quad 3 \quad \ldots \quad (k-2) \quad (k-1)\}_{\mathrm{RR}_{(\mathrm{n-k})}}$ having as last point [i], i.e. through all the routes $\mathrm{RERI}_{\mathrm{n}} \rightarrow \cdots \rightarrow \mathrm{RERI}_{\mathrm{n-i}} \rightarrow \mathrm{RERI}_{\mathrm{n-k}}$

To prove (A.SC.16), we have to show that from the total $\sum_{\binom{\mathrm{n}}{\mathrm{n-i}}} \mathrm{RR}_{(\mathrm{n-i})}$ of all different $\mathrm{RR}_{(\mathrm{n-i})}$, we end up to $\binom{\mathrm{k}}{\mathrm{i}} * \sum_{\binom{\mathrm{n}}{\mathrm{n-k}}} \mathrm{RR}_{(\mathrm{n-k})}$

different $\mathrm{RR}_{(\mathrm{n-k})}$.

We have that

$\binom{\mathrm{n}}{\mathrm{n-i}}$ different $\mathrm{RERI}_{\mathrm{n-i}}$'s:

- $\mathrm{RERI}_{\mathrm{n-i}}|X_1-, X_2-, \ldots, X_{i-1}-, X_i-$
- $\mathrm{RERI}_{\mathrm{n-i}}|X_2-, X_3-, \ldots, X_i-, X_{i+1}-$
- …

$\mathrm{RERI}_{\mathrm{n-k}}|X_1-, X_2-, \ldots, X_i-, \ldots, X_{k-1}-, X_k-$

$\mathrm{RERI}_{\mathrm{n-k}}|X_1-, X_2-, \ldots, X_i-, \ldots, X_{k-2}-, X_{k-1}-, X_{k+1}-$

…

$\binom{\mathrm{n-i}}{\mathrm{n-k}}$ different $\mathrm{RERI}_{\mathrm{n-k}}$'s created by $\mathrm{RERI}_{\mathrm{n-i}}|X_1-, X_2-, \ldots, X_{i-1}-, X_i-$

$\mathrm{RERI}_{\mathrm{n-k}}|X_1-, X_2-, \ldots, X_i-, \ldots, X_{k-1}-, X_k-$

$\mathrm{RERI}_{\mathrm{n-k}}|X_1-, X_2-, \ldots, X_i-, \ldots, X_{k-2}-, X_{k-1}-, X_{k+1}-$

…

$\binom{\mathrm{n-i}}{\mathrm{n-k}}$ different $\mathrm{RERI}_{\mathrm{n-k}}$'s created by $\mathrm{RERI}_{\mathrm{n-i}}|X_2-, X_3-, \ldots, X_i-, X_{i+1}-$

From above, we can observe that all the $\binom{n}{n-k}$ different $RERI_{n-k}$ are created from all the $\binom{n}{n-i}$ different $RERI_{n-i}$'s $\binom{k}{i}$ times. This is done because, for p∈P={1,.2,...,k}, $RERI_{n-k}|X_1 -, X_2 -, \dots, X_p -, \dots, X_k -$ is derived by all $RERI_{n-i}|i\ of\ the\ X_k = 0$, with i≤k, or in other words, i∈I⊂P, but from no other $RERI_{n-i}|any\ X_q -$, for q∉ P={1,.2,...,k}. The number of sets of I's , for which I⊂P is $\binom{k}{i}$. So the total $RR_{(n-k)}$ that will be created through the route $RERI_n \to \cdots \to RERI_{n-i} \to \cdots \to RERI_{n-k}$ is equal to $\sum_{\binom{n}{n-i}} \sum_{\binom{n-i}{n-k}} RR_{(n-k)} = \binom{k}{i} * \sum_{\binom{n}{n-k}} RR_{(n-k)}$. So (A.SC.16) holds.

Now, using (A.SC.16), we have that

$\{1\ \ 2\ \ 3\ \ \dots\ \ (k-2)\ \ \ (k-1)\}_{RR_{(n-k)}}$ is constructed by

$$\{1\ \ 2\ \ 3\ \ \dots\ \ (k-2)\ \ \ (k-1)\}_{RR_{(n-k);[k-1]}} = \sum_{\binom{n}{n-(k-1)}} \sum_{\binom{n-(k-1)}{n-k}} RR_{(n-k)} = \sum_{\binom{n}{n-k+1}} \sum_{\binom{n-k+1}{n-k}} RR_{(n-k)} = \binom{k}{k-1} \sum_{\binom{n}{n-k}} RR_{(n-k)}$$

$$\{1\ \ 2\ \ 3\ \ \dots\ \ (k-2)\ \ \ (k-1)\}_{RR_{(n-k);[k-2]}} = \sum_{\binom{n}{n-(k-2)}} \sum_{\binom{n-(k-2)}{n-k}} RR_{(n-k)} = \sum_{\binom{n}{n-k+2}} \sum_{\binom{n-k+2}{n-k}} RR_{(n-k)} = \binom{k}{k-2} \sum_{\binom{n}{n-k}} RR_{(n-k)}$$

...

$$\{1 \quad 2 \quad 3 \quad \dots \quad (k-2) \quad (k-1)\}_{\mathrm{RR}_{(n-k);[1]}} = \sum_{\binom{n}{n-(k-(k-1))}} \sum_{\binom{n-(k-(k-1))}{n-k}} \mathrm{RR}_{(n-k)} = \sum_{\binom{n}{n-1}} \sum_{\binom{n-1}{n-k}} \mathrm{RR}_{(n-k)} = \binom{k}{1} \sum_{\binom{n}{n-k}} \mathrm{RR}_{(n-k)}$$

$$\{1 \quad 2 \quad 3 \quad \dots \quad (k-2) \quad (k-1)\}_{\mathrm{RR}_{(n-k);[\emptyset]}} = \sum_{\binom{n}{n-(k-(k))}} \sum_{\binom{n-(k-(k))}{n-k}} \mathrm{RR}_{(n-k)} = \sum_{\binom{n}{n}} \sum_{\binom{n}{n-k}} \mathrm{RR}_{(n-k)} = \binom{k}{0} \sum_{\binom{n}{n-k}} \mathrm{RR}_{(n-k)}$$

and as we can see from (A.SC.13), in a way that

$$\begin{aligned} \{1 \quad 2 \quad 3 \quad \dots \quad (k-2) \quad (k-1)\}_{\mathrm{RR}_{(n-k)}} &= \{1 \quad 2 \quad 3 \quad \dots \quad (k-2) \quad (k-1)\}_{\mathrm{RR}_{(n-k);[k-1]}} \\ &\quad -\{1 \quad 2 \quad 3 \quad \dots \quad (k-2) \quad (k-1)\}_{\mathrm{RR}_{(n-k);[k-2]}} \\ &\quad +\{1 \quad 2 \quad 3 \quad \dots \quad (k-2) \quad (k-1)\}_{\mathrm{RR}_{(n-k);[k-3]}} \\ &\quad \dots \\ &\quad \pm\{1 \quad 2 \quad 3 \quad \dots \quad (k-2) \quad (k-1)\}_{\mathrm{RR}_{(n-k);[\emptyset]}} \end{aligned} \qquad \text{(A.SC.17)}$$

So extending (A.SC.14), we have that

$$\{1 \quad 2 \quad 3 \quad \dots \quad (k-2) \quad (k-1)\}_{\mathrm{RR}_{(n-k)}} = \sum_{j=1}^{k} (-1)^{(j-1)} \sum_{\binom{n}{n-(k-j)}} \sum_{\binom{n-(k-j)}{n-k}} \mathrm{RR}_{(n-k)}$$

So, as exactly we worked to prove (A.SC.7), we have that

$$\{1 \quad 2 \quad 3 \quad \ldots \quad (k-2) \quad (k-1)\}_{\mathrm{RR}_{(\mathrm{n-k})}} = \sum_{j=1}^{k}(-1)^{j-1}\binom{\mathrm{k}}{\mathrm{k}-(\mathrm{k}-\mathrm{j})}\sum_{\binom{\mathrm{n}}{\mathrm{n-k}}}\mathrm{RR}_{(\mathrm{n-k})} = \sum_{\binom{\mathrm{n}}{\mathrm{n-k}}}\mathrm{RR}_{(\mathrm{n-k})} * \sum_{j=1}^{k}(-1)^{j-1}\binom{\mathrm{k}}{\mathrm{j}}$$

$$= \sum_{\binom{\mathrm{n}}{\mathrm{n-k}}}\mathrm{RR}_{(\mathrm{n-k})} * \left(\binom{\mathrm{k}}{0} - \sum_{j=0}^{k}(-1)^{j}\binom{\mathrm{k}}{\mathrm{j}}\right)$$

$$= \sum_{\binom{\mathrm{n}}{\mathrm{n-k}}}\mathrm{RR}_{(\mathrm{n-k})} * \left(\binom{\mathrm{k}}{0} - \sum_{j=0}^{k}\binom{\mathrm{k}}{\mathrm{j}}(1)^{k-j} * (-1)^{j}\right)$$

$$= \sum\nolimits_{\binom{\mathrm{n}}{\mathrm{n-k}}}\mathrm{RR}_{(\mathrm{n-k})} * \left(1-(1-1)^{k}\right) = \sum\nolimits_{\binom{\mathrm{n}}{\mathrm{n-k}}}\mathrm{RR}_{(\mathrm{n-k})}$$

The transformation in the last line was applied because

$\sum_{j=0}^{k}\binom{\mathrm{k}}{\mathrm{j}}(x)^{k-j} * (y)^{j} = (x+y)^{k}$, for x=1, y=-1

Of note that the same hold for k=n-1 and k=n for $\mathrm{RR}_{(1)}$, $\mathrm{RR}_{(0)}$ respectively i.e. the total summation of $\mathrm{RR}_{(1)}$ and $\mathrm{RR}_{(0)}$ after replacing all $\mathrm{RERI}_{(\mathrm{n})}$ up to $\mathrm{RERI}_{(\mathrm{n-2})}$ from the recurrence relation (A.SC.3) and $\mathrm{ERR}_{(1)} = \mathrm{RR}_{(1)} - \mathrm{RR}_{(0)}$,

$$\sum_{j=1}^{n-1}(-1)^{j-1}\binom{\mathrm{n-1}}{\mathrm{n-1-(n-1-j)}}\sum_{\binom{\mathrm{n}}{\mathrm{n-(n-1)}}}\mathrm{RR}_{(\mathrm{n-(n-1)})}=\sum_{\binom{\mathrm{n}}{1}}\mathrm{RR}_{(1)}$$

and

$$\sum_{j=1}^{n}(-1)^{j-1}\binom{\mathrm{n}}{\mathrm{n-(n-j)}}\sum_{\binom{\mathrm{n}}{\mathrm{n-(n)}}}\mathrm{RR}_{(\mathrm{n-(n)})}=\mathrm{RR}_{(0)}$$

We also note from the (A.SC.17) that in the final solution, the sign changes from + to - (or vice versa) before each

$$\sum_{\binom{\mathrm{n}}{\mathrm{k}}}\mathrm{RR}_{(\mathrm{k})}$$

So we have that the final solution for the recurrence relationship (A.SC.3) is

$$\begin{aligned}\mathrm{RERI_n}(\mathrm{X_1,X_2,\dots,X_n})=\mathrm{RR}_{(\mathrm{n})}&\\ &-\sum_{\binom{\mathrm{n}}{\mathrm{n-1}}}\mathrm{RR}_{(\mathrm{n-1})}\\ &+\sum_{\binom{\mathrm{n}}{\mathrm{n-2}}}\mathrm{RR}_{(\mathrm{n-2})}\\ &\dots\end{aligned}$$

$$+(-1)^{n} * \sum_{\binom{n}{0}} RR_{(0)}$$

or in other words

$$RERI_{n}(X_{1}, X_{2}, \dots, X_{n}) = \sum_{k=0}^{n} \sum_{\binom{n}{n-k}} (-1)^{k} RR_{(n-k)} \qquad \boldsymbol{QED\ (see\ equation\ 6\ in\ the\ text)}$$

Proof of expression (6)

Connection between n-way interaction and (n-1) way interactions

We have to prove expression (7) from the paper, that is

$$\mathrm{RERI_n}(X_1, X_2, \dots, X_n) = \left(\mathrm{RERI}_{n-1}(X_1, X_2, \dots, X_n | X_i = 1) * \mathrm{RR}_{X_1 - X_2 - \dots X_{i+1} - X_i + X_{i+1} - \cdots X_{i+1} - \cdots X_n -}\right) - \mathrm{RERI}_{n-1}(X_1, X_2, \dots, X_n | X_i = 0)$$

From equation 6 in the paper, we have that

$$\mathrm{RERI_n}(X_1, X_2, \dots, X_n) = \mathrm{RR_{(n)}} - \sum_{\binom{n}{n-1}} \mathrm{RR_{(n-1)}} + \sum_{\binom{n}{n-2}} \mathrm{RR_{(n-2)}} - \sum_{\binom{n}{n-3}} \mathrm{RR_{(n-3)}} + \cdots + (-1)^n * \sum_{\binom{n}{0}} \mathrm{RR_{(0)}}$$

Using expression (6), we can calculate

$$\mathrm{RERI}_{n-1}(X_1, X_2, \dots, X_n | X_i = 1) = \frac{\mathrm{RR}_{(n);X_i+} - \sum_{\binom{n-1}{1}} \mathrm{RR}_{(n-1);X_i+} + \sum_{\binom{n-1}{2}} \mathrm{RR}_{(n-2);X_i+} \dots + (-1)^{n-1} * \sum_{\binom{n-1}{n-1}} \mathrm{RR}_{(1);X_i+}}{\mathrm{RR}_{X_1 - X_2 - \dots X_{i+1} - X_i + X_{i+1} - \cdots X_{i+1} - \cdots X_n}} \qquad \text{(A.SC.18)}$$

where $\mathrm{RR}_{(k);X_i+}$, k≤n and i≤n, is the relative risk, when k out of n risk factors are present and among them X_i is present as well.

In (A.SC.18), using the notation $\mathrm{RR}_{(k);X_i+}$, we denote the RR when k risk factors are present and n-k are absent and X_i is present

From the above, it is obvious that the term $\mathrm{RR}_{(1);X_i+}$ is equal to $\mathrm{RR}_{(1);X_i+} = \mathrm{RR}_{X_1 - X_2 - \dots X_{i+1} - X_i + X_{i+1} - \cdots X_{i+1} - \cdots X_n -}$

So, from (A.SC.18), we have that

$$\mathrm{RERI}_{n-1}(X_1, X_2, \dots, X_n | X_i = 1) * \mathrm{RR}_{X_1 - X_2 - \dots X_{i+1} - X_i + X_{i+1} - \cdots X_{i+1} - \cdots X_n -} =$$

$$= RR_{(n);X_i+} - \sum_{\binom{n-1}{1}} RR_{(n-1);X_i+} + \sum_{\binom{n-1}{2}} RR_{(n-2);X_i+} \dots + (-1)^{n-1} * \sum_{\binom{n-1}{n-1}} RR_{(1);X_i+} \qquad \text{(A.SC.19)}$$

In the same fashion, due to expression 6 we have that

$$RERI_{n-1}(X_1, X_2, \dots, X_n | X_i = 0) = RR_{(n-1);X_i-} - \sum_{\binom{n-1}{n-2}} RR_{(n-2);X_i-} + \sum_{\binom{n-1}{n-3}} RR_{(n-3);X_i-} \dots + (-1)^{n-1} * \sum_{\binom{n-1}{0}} RR_{(0);X_i-} \qquad \text{(A.SC.20)}$$

where $RR_{(k);X_i-}$, k≤n and i≤n, is the relative risk, when k out of n risk factors are present and risk factor X_i is absent.

Now, combining (A.SC.19) and (A.SC.20), we have that

$$\left(RERI_{n-1}(X_1, X_2, \dots, X_n | X_i = 1) * RR_{X_1-X_2-\dots X_{i+1}-X_i+X_{i+1}-\cdots X_{i+1}-\cdots X_n-}\right) - RERI_{n-1}(X_1, X_2, \dots, X_n | X_i = 0) =$$

$$= RR_{(n);X_i+} - \sum_{\binom{n-1}{1}} RR_{(n-1);X_i+} + \sum_{\binom{n-1}{2}} RR_{(n-2);X_i+} \dots + (-1)^{n-1} * \sum_{\binom{n-1}{n-1}} RR_{(1);X_i+}$$

$$-\left(RR_{(n-1);X_i-} - \sum_{\binom{n-1}{n-2}} RR_{(n-2);X_i-} + \sum_{\binom{n-1}{n-3}} RR_{(n-3);X_i-} \dots + (-1)^{n-1} * \sum_{\binom{n-1}{0}} RR_{(0);X_i-}\right)$$

Now if we try to summarize all $RR_{(n-j)}$ terms (1≤j≤n). We have that

$$\left(RERI_{n-1}(X_1, X_2, \dots, X_n | X_i = 1) * RR_{X_1-X_2-\dots X_{i+1}-X_i+X_{i+1}-\cdots X_{i+1}-\cdots X_n-}\right) - RERI_{n-1}(X_1, X_2, \dots, X_n | X_i = 0) = RR_{(n);X_i+}$$

$$-\left(\sum_{\binom{n-1}{n-2}} RR_{(n-1);X_i+} + RR_{(n-1);X_i-}\right)$$

$$+\left(\sum_{\binom{n-1}{n-3}} RR_{(n-1);X_i+} + \sum_{\binom{n-1}{n-2}} RR_{(n-1);X_i-}\right)$$

$$-\left(\sum_{\binom{n-1}{n-4}} RR_{(n-2);X_i+} + \sum_{\binom{n-1}{n-3}} RR_{(n-2);X_i-}\right)$$

$$\dots$$

$$+(-1)^j\left(\sum_{\binom{n-1}{n-j}} RR_{(n-j);X_i+} + \sum_{\binom{n-1}{n-(j-1)}} RR_{(n-j);X_i-}\right)$$

$$\dots$$

$$+(-1)^{n-2}\left(\sum_{\binom{n-1}{1}} RR_{(2);X_i+} + \sum_{\binom{n-1}{2}} RR_{(2);X_i-}\right)$$

$$+(-1)^{n-1}\left(\sum_{\binom{n-1}{0}} RR_{(1);X_i+} + \sum_{\binom{n-1}{1}} RR_{(1);X_i-}\right)$$

$$+(-1)^{n}\left(\sum_{\binom{n-1}{0}} RR_{(0);X_i-}\right) \quad \text{(A.SC.21)}$$

It is obvious from (A.SC.21), that if we prove for all $RR_{(n-j)}$ terms (1≤j≤n) that

$$+(-1)^j\left(\sum_{\binom{n-1}{n-j}} RR_{(n-j);X_i+} + \sum_{\binom{n-1}{n-(j-1)}} RR_{(n-j);X_i-}\right) = +(-1)^j\left(\sum_{\binom{n}{n-j}} RR_{(n-j)}\right) \quad \text{(A.SC.22)}$$

then we will have also proved equation (7) in the paper.

With a closer look at the first part of (A.SC.22), we have that $\sum_{\binom{n-1}{n-j}} RR_{(n-j);X_i+}$ and $\sum_{\binom{n-1}{n-(j-1)}} RR_{(n-j);X_i-}$ share no common RRs, given that $\sum_{\binom{n-1}{n-j}} RR_{(n-j);X_i+}$ is the summation of all the combinations of different RRs, when n-j risk factors are present and j are absent <u>and X_i is present</u>, while $\sum_{\binom{n-1}{n-(j-1)}} RR_{(n-j);X_i-}$ is the summation of all the combinations of different RRs, when n-j risk factors are present and j are absent <u>and X_i is absent</u>, so there is no overlapping of common RRs in that summation. Moreover, it is know from the Pascal rule that

$$\binom{n-1}{n-j} + \binom{n-1}{n-(j-1)} = \binom{n}{n-j}$$

So the summation of $\sum_{\binom{n-1}{n-j}} RR_{(n-j);X_i+}$ and $\sum_{\binom{n-1}{n-(j-1)}} RR_{(n-j);X_i-}$ will be equal to $\sum_{\binom{n}{n-j}} RR_{(n-j)}$, i.e. the summation of all the combinations of different RRs, when n-j risk factors are present and j are absent. ***QED***

So (A.SC.22) is proven, which means that expression (7) in the paper is proven as well.

Section D - Clarifications and recommendations for calculating multi-way interactions

When dealing only with two risk factors, the definition of RERI is pretty straightforward. The only issue that we should take into consideration is that RERI is calculated by risk and not protective factors. So if any of the binary variables Z_1 or Z_2 we use in the analysis is a protective factor, we need to recode them to a risk factor. For example, if Z_1 and Z_2 are both protective factors, we need to create variables $X_1=1-Z_1$ and $X_2=1-Z_2$ and calculate RERI(X_1,X_2). Nevertheless, when it is of interest the calculation of higher order interactions on the additive scale, we showed that the definitions are getting more complex, so we will try to further elaborate on them.

Three-way interactions

To shed more light in the properties of joint effects of the 3 risk factors of interest, we propose the following steps;

1) Conduct the analysis with the exposure of interest Z_1, Z_2, Z_3, without using any interaction term. We run e.g. Cox regression, and we check if the hazard ratios (HR) are greater than one (i.e. if the exposures are risk factors). If, for example, we have that HR(Z_1)>1, HR(Z_2)>1, but HR(Z_3)<1, (i.e. Z_1, Z_2 are risk factors, but Z_3 is protective), we have to use the variables $X_1=Z_1$, $X_2=Z_2$, and $X_3=1-Z_3$. In our illustrative paradigm, we didn't use a variable for the adherence to MD, which is a protective factor for mortality, bur for non-adherence to MD instead.

2) Perform the analysis with the risk factors of interest X_1, X_2, X_3, this time using the interaction terms X_1X_2, X_1X_3, X_2X_3 and $X_1X_2X_3$, as presented in model (A.SB.1) in the Appendix (Section B)

3) Compute $TotRERI_3(X_1,X_2,X_3)$ (from A.SB.2, Appendix, Section B) and find out whether the effects of X_1, X_2, X_3 are super-additive ($TotRERI_3(X_1,X_2,X_3)>0$), additive ($TotRERI_3(X_1,X_2,X_3)=0$) or sub-additive ($TotRERI_3(X_1,X_2,X_3)<0$). In our example, we show that the effects of low MD, obesity, and current smoking are super-additive ($TotRERI_3(X_1,X_2,X_3)=1.20>0$).

4) Estimate $RERI_3(X_1,X_2,X_3)$ (from A.SB.3, Appendix, Section B) to check whether any deviation from additivity of the three risk factors ($TotRERI_3(X_1,X_2,X_3)$) is attributed to the 3-way interaction, beyond the two way interactions, given that the 3rd risk factor is absent [see equation (3)]. In our example, there was evidence for 3-way additive interaction of the risk factors, beyond their two way interactions.

5) Calculate $RERI_2(X_1,X_2|X_3=0)$, $RERI_2(X_1,X_3|X_2=0)$ and $RERI_2(X_2,X_3|X_1=0)$ [from (A.SB.4) - (A.SB.6), Appendix, Section B) to test whether any deviation from additivity of the three risk factors (expressed through $TotRERI_3(X_1,X_2,X_3)$) is attributed to additive interaction of the two risk factors [(see equation (3)], when the third is absent. In our paradigm, all $RERI_2$ given the absence of the 3rd risk factor were negative, but of small magnitude and not statistically significant.

6) Compute $RERI_2(X_1,X_2|X_3=1)$, $RERI_2(X_1,X_3|X_2=1)$ and $RERI_2(X_2,X_3|X_1=1)$ (from (A.SB.7) – (A.SB.9) in Appendix, Section B) to check to what extend the interaction due to three risk factors exclusively (i.e $RERI_3(X_1,X_2,X_3)$) can be interpreted as interaction of the two risk factors, given that the 3rd is present. If we combine that information with

the one from step 5, we can additionally check whether $RERI_2$'s remain constant across the strata of the 3rd risk factor of interest. In our paradigm, we observed that all $RERI_2$ were positive, given the presence of the 3rd risk factor. If we additionally take into consideration $RERI_2$'s given the absence of the 3rd risk factor (from the previous step), we draw the conclusion that there is difference in how two of these variables interact across the levels of the 3rd factor.

7) Check for qualitative interaction, i.e. whether

a) the risk of X_1 is increasing across the strata of X_2 and X_3, i.e.

$RR_{X_1+X_2+X_3+} > RR_{X_1-X_2+X_3+}$, $RR_{X_1+X_2+X_3-} > RR_{X_1-X_2+X_3-}$,

$RR_{X_1+X_2-X_3+} > RR_{X_1-X_2-X_3+}$ and $RR_{X_1+X_2-X_3-} > RR_{X_1-X_2-X_3-}$

b) the risk of X_2 is increasing across the strata of X_1 and X_3, i.e.

$RR_{X_1+X_2+X_3+} > RR_{X_1+X_2-X_3+}$, $RR_{X_1+X_2+X_3-} > RR_{X_1+X_2-X_3-}$,

$RR_{X_1-X_2+X_3+} > RR_{X_1-X_2-X_3+}$ and $RR_{X_1-X_2+X_3-} > RR_{X_1-X_2-X_3-}$

c) the risk of X_3 is increasing across strata of X_1 and X_2, i.e.

$RR_{X_1+X_2+X_3+} > RR_{X_1+X_2+X_3-}$, $RR_{X_1+X_2-X_3+} > RR_{X_1+X_2-X_3-}$,

$RR_{X_1-X_2+X_3+} > RR_{X_1-X_2+X_3-}$ and $RR_{X_1-X_2-X_3+} > RR_{X_1-X_2-X_3-}$

For more details on qualitative interaction, please see Section F

<u>n-way interactions</u>

The total relative excess risk due to interaction ($TotRERI_n$) is calculated by comparing the joint effect of all n risk factors to the situation when each one acts separately (expression 5). However, $TotRERI_n$ is affected by all lower-order interactions of the n risk factors and not

exclusively by the n-way interaction of the risk factors. From equation (A.SC.2), we observe that the sign and magnitude of $TotRERI_n$ depends on the sign and magnitude of $\sum_{\binom{n}{n-1}} RERI_{n-1}(X_1, X_2, \ldots, X_n | 1 \text{ of the } X_i = 0)$, $\sum_{\binom{n}{n-2}} RERI_{n-2}(X_1, X_2, \ldots, X_n | 2 \text{ of the } X_i = 0)$, …, $\sum_{\binom{n}{2}} RERI_2(X_1, X_2, \ldots, X_n | (n-2) \text{ of the } X_i = 0)$. So, $RERI_n(X_1, X_2, \ldots, X_n)$ measures the interaction between n risk factors on the additive scale, as this index does not account for all the lower order additive interactions. To shed more light in the properties of joint effects of the n risk factors, we propose the following steps;

1) Apply step 1 as in recommendations in the 3-way interactions section, this time for Z_1, Z_2, …, Z_n. From this step, we will end up with the risk factors X_1, X_2, …, X_n.
2) Include all possible 2,3,…,n product terms constructed by X_1, X_2, …, X_n in the model, as described in step 2 in recommendations in the 3way interactions section.
3) Calculate $TotRERI_n(X_1, X_2, \ldots, X_n)$ from expression (5).
4) Calculate $RERI_n(X_1, X_2, \ldots, X_n)$ from equation (6)
5) Compute all $RERI_{n-1}$ given the 1 risk factor is absent ($RERI_{n-1}(X_1, X_2, \ldots, X_n | 1 \text{ of the } X_i = 0)$) from expression (6).
6) Estimate all $RERI_{n-1}$ given the 1 risk factor is present ($RERI_{n-1}(X_1, X_2, \ldots, X_n | 1 \text{ of the } X_i = 1)$) from equation (A.SC.19)
7) We can additionally compute all $TotRERI_{n-k}$ and $RERI_{n-k}$, $2 \leq k \leq n-2$, given k risk factors are all absent ($TotRERI_{n-k}(X_1, X_2, \ldots, X_n | k \text{ of the } X_i = 0)$ and $RERI_{n-k}(X_1, X_2, \ldots, X_n | k \text{ of the } X_i = 0)$) to understand how and under which conditions the n-k risk factors interact for the development of a specific disease. On the same fashion, we can compute all $TotRERI_{n-k}(X_1, X_2, \ldots, X_n | k \text{ of the } X_i = 1)$ and $RERI_{n-k}(X_1, X_2, \ldots, X_n | k \text{ of the } X_i = 1)$

8) Check for qualitative interaction, that is

whether the risk of X_1 is increasing across the strata of X_2, X_3 … X_n,.

whether the risk of X_2 is increasing across the strata of X_1, X_3 … X_n,

…

whether the risk of X_n is increasing across the strata of X_1, X_2 … X_{n-1}

Section E – Multiplicative interaction and its connection to additive interaction

2-way interactions

The usual practice of the researchers is to refer to statistical interaction when studying interaction between risk factors. Nevertheless, under this concept, interaction is measured on either additive or multiplicative scale, depending on the form of the underlying model used. It is known in the study of the 2-way interactions that then we use Cox or logistic regression, which are inherently multiplicative models, then the beta coefficient of the product term shows whether there is any deviation from the multiplicativity of the effects of two risk factors. More specifically, the effects are super- or sub-multiplicative, if the beta coefficient is greater or lower than zero respectively (or, equivalently, if the odds/hazard ratio is higher or lower than 1). For example, if we run a Cox regression model with exposures X_1 and X_2, i.e.

$$\lambda(t) = \lambda_0(t) * \exp(b_1X_1 + b_2X_2 + b_3X_1X_2)\,, \quad \text{(A.SE.1)}$$

then, we calculate

$$HR(X_1 = 0, X_2 = 0) = RR_{X_1-X_2-} = 1$$

$$HR(X_1 = 1, X_2 = 0) = RR_{X_1+X_2-} = \exp(b_1)$$

$$HR(X_1 = 0, X_2 = 1) = RR_{X_1-X_2+} = \exp(b_2)$$

$$HR(X_1 = 1, X_2 = 1) = RR_{X_1+X_2+} = \exp(b_1 + b_2 + b_3)$$

Then the multiplicative interaction

$$I_2 = \frac{RR_{X_1+X_2+}}{RR_{X_1+X_2-} * RR_{X_1-X_2+}} = \exp(b_3)\,, \qquad \text{(A.SE.2)}$$

So from (A.SE.2), the effects are super-multiplicative ($I_2>1$), if $b_3>0$,

the effects are multiplicative (I_2=1), if b_3=0 and

the effects are sub-multiplicative (I_2<1), if b_3<0,

that is, the statistical interaction will show whether there is any deviation from multiplicativity of the effects.

Regarding the connection between multiplicative and additive 2-way interaction, there are 2 inequalities that link the deviation from additivity and from multiplicativity. Both of them hold when there is no qualitative interaction. They also hold if we relax the assumptions of qualitative interaction and we assume only that $RR_{X_1+X_2-} > RR_{X_1-X_2-}(=1)$ and

$RR_{X_1-X_2+} > RR_{X_1-X_2-}(=1)$

1) When the effects of 2 risk factors are either multiplicative of super-multiplicative, then the effects will be super-additive.

Proof: We have that

$$RERI_2(X_1, X_2) = RR_{X_1+X_2+} - RR_{X_1+X_2-} - RR_{X_1-X_2+} + 1 \quad , \quad \text{(A.SE.3)}$$

In the case of multiplicative or super-multiplicative effects of X_1 and X_2, i.e. when $I_2 \geq 1$, from (A.SE.2), $RR_{X_1+X_2+} \geq RR_{X_1+X_2-} * RR_{X_1-X_2+}$, so from (A.SE.3) we have

$$\begin{aligned} RERI_2(X_1, X_2) &\geq RR_{X_1+X_2-} * RR_{X_1-X_2+} - RR_{X_1+X_2-} - RR_{X_1-X_2+} + 1 \\ &= RR_{X_1+X_2-} * \left(RR_{X_1-X_2+} - 1\right) - \left(RR_{X_1-X_2+} - 1\right) \\ &= \left(RR_{X_1+X_2-} - 1\right) * \left(RR_{X_1-X_2+} - 1\right) > 0 \end{aligned}$$

because $RR_{X_1+X_2-}$ and $RR_{X_1+X_2-} > 1$, as X_1 and X_2 are risk factors. In other words, we proved that when the effects of 2 risk actors are either multiplicative of super-multiplicative, then the effects will be super-additive.

2) When the effects of 2 risk factors are either additive or sub-additive, then the effects will be sub-multiplicative.

Proof: If $RERI_2(X_1, X_2) \leq 0$, then $RR_{X_1+X_2+} \leq RR_{X_1+X_2-} + RR_{X_1-X_2+} - 1$, so

$$\frac{RR_{X_1+X_2+}}{RR_{X_1+X_2-}+RR_{X_1-X_2+}-1} \leq 1 \quad \text{(A.SE.4)},$$

so we have to prove that

$RR_{X_1+X_2-} + RR_{X_1-X_2+} - 1 < RR_{X_1+X_2-} * RR_{X_1-X_2+}$, or equivalently

$RR_{X_1+X_2-} * RR_{X_1-X_2+} - RR_{X_1+X_2-} - RR_{X_1-X_2+} + 1 > 0$ or equivalently

$(RR_{X_1+X_2-} - 1) * (RR_{X_1-X_2+} - 1) > 0$, which is true

So from (A.SE.4), we have that

$$I_2 = \frac{RR_{X_1+X_2+}}{RR_{X_1+X_2-} * RR_{X_1-X_2+}} < \frac{RR_{X_1+X_2+}}{RR_{X_1+X_2-} + RR_{X_1-X_2+} - 1} \leq 1$$

So, $I_2<1$. In other words, we proved that when the effects of 2 risk factors are either additive or sub-additive, then the effects will be sub-multiplicative.

<u>3-way interactions</u>

In case of study of 3-way interaction on the multiplicative scale, we should extend the definitions to three risk factors X_1, X_2 and X_3. We will use the worked example from our paper, that is we will use the Cox regression model (A.SB.1).

For the deviation from multiplicativity of the effects of these factors, one should compare

$$RR_{X_1+X_2+X_3+} \quad \text{vs} \quad RR_{X_1+X_2-X_3-} * RR_{X_1-X_2+X_3-} * RR_{X_1-X_2-X_3+}$$

So, we can calculate $TotI_3(X_1, X_2, X_3)$ to check if there is any deviation from multiplicativity

$$\text{TotI}_3(X_1, X_2, X_3) = \frac{RR_{X_1+X_2+X_3+}}{RR_{X_1+X_2-X_3-} * RR_{X_1-X_2+X_3-} * RR_{X_1-X_2-X_3+}} \quad \text{(A.SE.5)}$$

So we have that

$$\text{TotI}_3(X_1, X_2, X_3) = \frac{\exp(a_1+a_2+a_3+a_4+a_5+a_6+a_7)}{\exp(a_1)*\exp(a_2)*\exp(a_3)} = \exp(a_4 + a_5 + a_6 + a_7) \quad \text{(A.SE.6)}$$

On the same fashion, the 2-way multiplicative interaction, given the third factor is absent will be

$$I_2(X_1, X_2 | X_3 = 0) = \frac{\exp(a_1+a_2+a_4)}{\exp(a_1)*\exp(a_2)} = \exp(a_4) \quad \text{(A.SE.7)}$$

$$I_2(X_1, X_3 | X_2 = 0) = \frac{\exp(a_1+a_3+a_5)}{\exp(a_1)*\exp(a_3)} = \exp(a_5) \quad \text{(A.SE.8)}$$

$$I_2(X_2, X_3 | X_1 = 0) = \frac{\exp(a_2+a_3+a_6)}{\exp(a_1)*\exp(a_2)} = \exp(a_6) \quad \text{(A.SE.9)}$$

Moreover, the 2-way multiplicative interaction, given the third factor is present will be

$$I_2(X_1, X_2 | X_3 = 1) = \frac{\exp(a_1+a_2+a_3+a_4+a_5+a_6+a_7)/\exp(a_3)}{(\frac{\exp(a_1+a_3+a_5)}{\exp(a_3)})*(\frac{\exp(a_2+a_3+a_6)}{\exp(a_3)})} = \exp(a_4 + a_7) \quad \text{(A.SE.10)}$$

$$I_2(X_1, X_3 | X_2 = 1) = \frac{\exp(a_1+a_2+a_3+a_4+a_5+a_6+a_7)/\exp(a_2)}{(\frac{\exp(a_1+a_2+a_4)}{\exp(a_2)})*(\frac{\exp(a_2+a_3+a_6)}{\exp(a_2)})} = \exp(a_5 + a_7) \quad \text{(A.SE.11)}$$

$$I_2(X_2, X_3 | X_1 = 1) = \frac{\exp(a_1+a_2+a_3+a_4+a_5+a_6+a_7)/\exp(a_1)}{(\frac{\exp(a_1+a_2+a_4)}{\exp(a_1)})*(\frac{\exp(a_1+a_3+a_5)}{\exp(a_1)})} = \exp(a_6 + a_7) \quad \text{(A.SE.12)}$$

and the 3-way multiplicative interaction, beyond the 2-way interactions will be

$$I_3(X_1, X_2, X_3) = \frac{\text{TotI}_3(X_1, X_2, X_3)}{I_2(X_1, X_2 | X_3 = 0) * I_2(X_1, X_3 | X_2 = 0) * I_2(X_2, X_3 | X_1 = 0)}$$

And if we make the calculations

$$I_3(X_1, X_2, X_3) = \exp(a_7) \quad \text{(A.SE.13)}$$

n-way interactions

By extending the definitions of checking for deviation from multiplicativity to the n-way interactions, we have that one should compare

$RR_{X_1+X_2+\cdots X_n+}$ vs $RR_{X_1+X_2-\cdots X_n-} * RR_{X_1-X_2+X_3-\cdots X_n-} * \ldots * RR_{X_1-X_2-\cdots X_{n-1}-X_n+}$

We can calculate $\mathrm{TotI}_n(X_1, X_2, \ldots, X_n)$ to check if there is any deviation from multiplicativity

$$\mathrm{TotI}_n(X_1, X_2, \ldots, X_n) = \frac{RR_{X_1+X_2+\cdots X_n+}}{RR_{X_1+X_2-\cdots X_n-} * RR_{X_1-X_2+X_3-\cdots X_n-} * \ldots * RR_{X_1-X_2-\cdots X_{n-1}-X_n+}} \quad \text{(A.SE.14)}$$

To check deviation from additivity, we have from equation (5) that we have to check

$$\mathrm{TotRERI}_n(X_1, X_2, \ldots, X_n) = RR_{X_1+X_2+\cdots X_n+} - RR_{X_1+X_2-\cdots X_n-}$$
$$-RR_{X_1+X_2-\cdots X_n-} - \cdots - RR_{X_1-X_2-\cdots X_{n-1}-X_n+} + (n-1) \quad \text{(A.SE.15)}$$

We will generalize the inequalities we showed for 2-way interactions to the n-way interactions. Both of them hold when there is no qualitative interaction. They also hold if we relax the assumptions of qualitative interaction and we assume only that

$RR_{X_1+X_2-\cdots X_n-} > RR_{X_1-X_2-\cdots X_n-}(=1)$ and

$RR_{X_1-X_2+X_3-\cdots X_n-} > RR_{X_1-X_2-\cdots X_n-}(=1)$ and

…

$RR_{X_1-X_2-\cdots X_{n-1}-X_n+} > RR_{X_1-X_2-\cdots X_n-}(=1)$

More specifically, we will prove

1) When the effects of n risk factors are either multiplicative of super-multiplicative, then the effects will be super-additive.

Proof: If $\mathrm{TotI}_n(X_1, X_2, \ldots, X_n) \geq 1$, we have that

$$\text{TotRERI}_n(X_1, X_2, \dots, X_n) \geq RR_{X_1+X_2-\cdots X_n-} * \ RR_{X_1-X_2+X_3-\cdots X_n-} \ * \dots * \ RR_{X_1-X_2-\cdots X_{n-1}-X_n+}$$

$$-RR_{X_1+X_2-\cdots X_n-} - RR_{X_1-X_2+X_3-\cdots X_n-} - \cdots - RR_{X_1-X_2-\cdots X_{n-1}-X_n+} + (n-1)$$

Now we have to prove that

$$RR_{X_1+X_2-\cdots X_n-} * \ RR_{X_1-X_2+X_3-\cdots X_n-} \ * \dots * \ RR_{X_1-X_2-\cdots X_{n-1}-X_n+}$$

$$-RR_{X_1+X_2-\cdots X_n-} - RR_{X_1-X_2+X_3-\cdots X_n-} - \cdots - RR_{X_1-X_2-\cdots X_{n-1}-X_n+} + (n-1) > 0 \qquad \text{(A.SE.16)}$$

We have that

$$RR_{X_1+X_2-\cdots X_n-} * \ RR_{X_1-X_2+X_3-\cdots X_n-} \ * \dots * \ RR_{X_1-X_2-\cdots X_{n-1}-X_n+}$$

$$-RR_{X_1+X_2-\cdots X_n-} - RR_{X_1-X_2+X_3-\cdots X_n-} - \cdots - RR_{X_1-X_2-\cdots X_{n-1}-X_n+} + (n-1) =$$

1st step → remove $RR_{X_1+X_2-\cdots X_n-}$ from the equation

$$= RR_{X_1+X_2-\cdots X_n-} * \ (RR_{X_1-X_2+X_3-\cdots X_n-} \ * \dots * \ RR_{X_1-X_2-\cdots X_{n-1}-X_n+} - 1)$$

$$-RR_{X_1-X_2+\cdots X_n-} - \cdots - RR_{X_1-X_2-\cdots X_{n-1}-X_n+} + (n-1) > \qquad \text{(because } RR_{X_1+X_2-\cdots X_n-} > 1)$$

$$(RR_{X_1-X_2+X_3-\cdots X_n-} \ * \dots * \ RR_{X_1-X_2-\cdots X_{n-1}-X_n+} - 1)$$

$$-RR_{X_1-X_2+\cdots X_n-} - \cdots - RR_{X_1-X_2-\cdots X_{n-1}-X_n+} + (n-1) =$$

$$(RR_{X_1-X_2+X_3-\cdots X_n-} \ * \dots * \ RR_{X_1-X_2-\cdots X_{n-1}-X_n+})$$

$$-RR_{X_1-X_2+\cdots X_n-} - \cdots - RR_{X_1-X_2-\cdots X_{n-1}-X_n+} + (n-2) =$$

2nd step → remove $RR_{X_1-X_2+X_3-\cdots X_n-}$ from the equation

$$= RR_{X_1-X_2+X_3-\cdots X_n-} * \ (RR_{X_1-X_2-X_3+X_4-\cdots X_n-} \ * \dots * \ RR_{X_1-X_2-\cdots X_{n-1}-X_n+} - 1)$$

$$-RR_{X_1-X_2-X_3+X_4-\cdots X_n-} - \cdots - RR_{X_1-X_2-\cdots X_{n-1}-X_n+} + (n-2) > \qquad \text{(because } RR_{X_1-X_2+X_3-\cdots X_n-} > 1)$$

$$(RR_{X_1-X_2-X_3+X_4-\cdots X_n-} \ * \dots * \ RR_{X_1-X_2-\cdots X_{n-1}-X_n+} - 1)$$

$$-RR_{X_1-X_2+\cdots X_n-} - \cdots - RR_{X_1-X_2-\cdots X_{n-1}-X_n+} + (n-2) =$$

$(RR_{X_1-X_2-X_3+X_4-\cdots X_n-} * \ldots * RR_{X_1-X_2-\cdots X_{n-1}-X_n+})$

$-RR_{X_1-X_2+\cdots X_n-} - \cdots - RR_{X_1-X_2-\cdots X_{n-1}-X_n+} + (n-3) >$

…

(n-2)th step → remove $RR_{X_1-X_2-\cdots X_{n-3}-X_{n-2}+X_{n-1}-X_n-}$ from the equation)

$RR_{X_1-X_2-\cdots X_{n-3}-X_{n-2}+X_{n-1}-X_n-} * (RR_{X_1-X_2-\cdots X_{n-2}-X_{n-1}+X_n-} * RR_{X_1-X_2-\cdots X_{n-1}-X_n+} - 1)$

$-RR_{X_1-X_2-\cdots X_{n-2}-X_{n-1}+X_n-} - RR_{X_1-X_2-\cdots X_{n-1}-X_n+} + (n-(n-2)) >$

(because $RR_{X_1-X_2-\cdots X_{n-3}-X_{n-2}+X_{n-1}-X_n-} > 1$)

$(RR_{X_1-X_2-\cdots X_{n-2}-X_{n-1}+X_n-} * RR_{X_1-X_2-\cdots X_{n-1}-X_n+} - 1)$

$-RR_{X_1-X_2-\cdots X_{n-2}-X_{n-1}+X_n-} - RR_{X_1-X_2-\cdots X_{n-1}-X_n+} + \left(n-(n-2)\right) =$

$(RR_{X_1-X_2-\cdots X_{n-2}-X_{n-1}+X_n-} * RR_{X_1-X_2-\cdots X_{n-1}-X_n+})$

$-RR_{X_1-X_2-\cdots X_{n-2}-X_{n-1}+X_n-} - RR_{X_1-X_2-\cdots X_{n-1}-X_n+} + 2 - 1 =$

$(RR_{X_1-X_2-\cdots X_{n-2}-X_{n-1}+X_n-} * RR_{X_1-X_2-\cdots X_{n-1}-X_n+})$

$-RR_{X_1-X_2-\cdots X_{n-2}-X_{n-1}+X_n-} - RR_{X_1-X_2-\cdots X_{n-1}-X_n+} + 1 =$

$\left(RR_{X_1-X_2-\cdots X_{n-2}-X_{n-1}+X_n-}\right) * \left(RR_{X_1-X_2-\cdots X_{n-1}-X_n+} - 1\right) - (RR_{X_1-X_2-\cdots X_{n-1}-X_n+} - 1) =$

$\left(RR_{X_1-X_2-\cdots X_{n-2}-X_{n-1}+X_n-} - 1\right) * \left(RR_{X_1-X_2-\cdots X_{n-1}-X_n+} - 1\right) > 0$

because $RR_{X_1-X_2-\cdots X_{n-2}-X_{n-1}+X_n-}$ *and* $RR_{X_1-X_2-\cdots X_{n-1}-X_n+} > 1$

So, we proved that when the effects of n risk factors are either multiplicative of super-multiplicative, then the effects will be super-additive.

2) When the effects of n risk factors are either additive or sub-additive, then the effects will be sub-multiplicative.

Proof: If $\text{TotRERI}_n(X_1, X_2, \dots, X_n) \le 0$, then

$$RR_{X_1+X_2-\cdots X_n-} * \; RR_{X_1-X_2+X_3-\cdots X_n-} \; * \dots * \; RR_{X_1-X_2-\cdots X_{n-1}-X_n+}$$

$$-RR_{X_1+X_2-\cdots X_n-} - RR_{X_1-X_2+X_3-\cdots X_n-} - \cdots - RR_{X_1-X_2-\cdots X_{n-1}-X_n+} + (n-1) \le 0$$

or equivalently

$$\frac{RR_{X_1+X_2-\cdots X_n-} * RR_{X_1-X_2+X_3-\cdots X_n-} * \dots * RR_{X_1-X_2-\cdots X_{n-1}-X_n+}}{RR_{X_1+X_2-\cdots X_n-} + RR_{X_1-X_2+X_3-\cdots X_n-} + \cdots + RR_{X_1-X_2-\cdots X_{n-1}-X_n+} - (n-1)} \le 1 \quad \text{(A.SE.17)}$$

From (A.SE.14), we have that

$$\text{TotI}_n(X_1, X_2, \dots, X_n) = \frac{RR_{X_1+X_2+\cdots X_n+}}{RR_{X_1+X_2-\cdots X_n-} * \; RR_{X_1-X_2+X_3-\cdots X_n-} \; * \dots * \; RR_{X_1-X_2-\cdots X_{n-1}-X_n+}} <$$

(because of (A.SE.16))

$$\frac{RR_{X_1+X_2-\cdots X_n-} * RR_{X_1-X_2+X_3-\cdots X_n-} * \dots * RR_{X_1-X_2-\cdots X_{n-1}-X_n+}}{RR_{X_1+X_2-\cdots X_n-} + RR_{X_1+X_2-\cdots X_n-} + \cdots + RR_{X_1-X_2-\cdots X_{n-1}-X_n+} - (n-1)} \le 1 \quad \text{because of (A.SE.17)}$$

So we proved that when $\text{TotRERI}_n(X_1, X_2, \dots, X_n) \le 0$, then $\text{TotI}_n(X_1, X_2, \dots, X_n) < 1$. In other words, we showed that when the effects of n risk factors are either additive or sub-additive, then the effects will be sub-multiplicative.

<u>Connection between deviation from additivity and multiplicativity from the worked example</u>

We can calculate TotI_3=1.20>1 from (A.SE.6), so the effects of low MD, obesity and smoking status on mortality are super-multiplicative (even not statistically significant), meaning that we also expect that these effects would also be super-additive (from the 1st inequality). This is true, because TotRERI_3=1.20>0, in other words, there is an excess 120% risk due to the joint presence of all risk factors, compared to the situation that each of them would act separately

The 3-way interaction of these factors beyond the 2-way interactions was positive both under the additive and under the multiplicative scale ($RERI_3$=1.98 and I_3=2.51), however there is not a direct link between these two indeces. The only conclusion that we can infer from equations (A.SB3) and (A.SE13) is that the greater the value of I_3 is, the greater the value of $RERI_3$ will be as well (the opposite is not always true), without any guarantee that $RERI_3$ will be positive, depending on a specific value for I_3.

Moreover, all the $RERI_2$ given the absence of the 3rd risk factor are negative, indicating that the corresponding all the effects between 2 risk factors, when the 3rd is absent, will be sub-multiplicative (from the 2nd inequality). More specifically, we had that

I_2(low MD, high BMI / never or former smokers)=0.77<1

I_2(low MD, current smokers / low BMI)= 0.79<1

I_2(high BMI, current smokers / high MD)= 0.79<1

Regarding the 2-way interactions, when the 3rd factor was present, when we calculate that all the I_2's, we find that the effects of every 2 risk factors, when the 3rd is present, were super-multiplicative;

I_2(low MD, high BMI / current smokers)=1.98>1

I_2(low MD, current smokers / high BMI)= 1.99>1

I_2(high BMI, current smokers / low MD)= 1.92>1

indicating that the corresponding effects will be super-additive, that's why all $RERI_2$'s were positive.

In this example, we found that the interpretation doesn't change, if we convert to deviation from multiplicativity as reference. However, this is not always true. We showed above that

1) If $TotI_3 \geq 1 \rightarrow TotRERI_3 > 0$ and

2) If $TotRERI_3 \leq 0 \rightarrow TotI_3 < 1$

The opposite in these relationships does not always hold. In other words, it is possible to observe super-additive effects, which can be sub-multiplicative ($TotRERI_3>0$ & $TotI_3<1$)

We also mentioned that the greater the value of I_3 is, the greater the value of $RERI_3$ will be. Nevertheless, there is no specific interval lower limit for $RERI_3$ for different values of I_3.

Section F – Qualitative interaction

We refer to the term qualitative (or cross-over) interaction when the exposure of interest is a risk factor for a specific outcome for one subgroup, but a protective factor for another subgroup. For example, a specific medication might be beneficial in younger people, but not in the elderly. Qualitative interaction is very crucial for decision making for public health purposes, because, in such instances, we should not treat all the subgroups, but only those people for which the medication is beneficial.

To check for qualitative interaction in case of 2 risk factors X_1 and X_2, we should check

1) Whether the risk of X_1 is increasing across the strata of X_2, i.e.

$RR_{X_1+X_2+} > RR_{X_1-X_2+}$ and $RR_{X_1+X_2-} > RR_{X_1-X_2-}$

and

2) Whether the risk of X_2 is increasing across the strata of X_1, i.e.

$RR_{X_1+X_2+} > RR_{X_1+X_2-}$ and $RR_{X_1-X_2+} > RR_{X_1-X_2-}$

To apply the same in case of 3 risk factors X_1, X_2 and X_3, we should check

1) whether the risk of X_1 is increasing across the strata of X_2 and X_3, i.e.

$RR_{X_1+X_2+X_3+} > RR_{X_1-X_2+X_3+}$, $RR_{X_1+X_2+X_3-} > RR_{X_1-X_2+X_3-}$, $RR_{X_1+X_2-X_3+} > RR_{X_1-X_2-X_3+}$ and $RR_{X_1+X_2-X_3-} > RR_{X_1-X_2-X_3-}$

2) whether the risk of X_2 is increasing across the strata of X_1 and X_3, i.e.

$RR_{X_1+X_2+X_3+} > RR_{X_1+X_2-X_3+}$, $RR_{X_1+X_2+X_3-} > RR_{X_1+X_2-X_3-}$, $RR_{X_1-X_2+X_3+} > RR_{X_1-X_2-X_3+}$ and $RR_{X_1-X_2+X_3-} > RR_{X_1-X_2-X_3-}$

3) whether the risk of X_3 is increasing across strata of X_1 and X_2, i.e.

$RR_{X_1+X_2+X_3+} > RR_{X_1+X_2+X_3-}$, $RR_{X_1+X_2-X_3+} > RR_{X_1+X_2-X_3-}$, $RR_{X_1-X_2+X_3+} > RR_{X_1-X_2+X_3-}$ and $RR_{X_1-X_2-X_3+} > RR_{X_1-X_2-X_3-}$

The same procedure should be followed for multi-way interactions, i.e. to check

1) whether the risk of X_1 is increasing across the strata of X_2, X_3 … X_n,.

2) whether the risk of X_2 is increasing across the strata of X_1, X_3 … X_n,

…

n) whether the risk of X_n is increasing across the strata of X_1, X_2 … X_{n-1}

In Section C of the Appendix, we show how these calculations can be applied in Stata, in case of 3 risk factors, X_1, X_2 and X_3.

Qualitative interaction occurs very rarely in the study of the joint effects of 2 risk factors, that's why it is not mentioned in the literature frequently. It is very likely to happen rarely in the case of 3 risk factors as well, but it is more possible to occur when studying more >3 risk factors (the more we increase the factors of interest, the more likely to observe qualitative interactions)
So, when conducting a multi-way interaction analysis, we should additionally test whether the risk of a risk factor, is increasing across the different subgroups of interest of the population.
In other words, a researcher should assess the results from the interaction analysis (TotRERI and RERIs), as we did in the main body of the manuscript, and additionally test whether there is qualitative interaction. In the case that qualitative interaction exists, then one should comment on the corresponding consequences (e.g. that a specific medication is protective for CVD in one

subgroup of the study while it is not in another), apart from the discussion of the results of interaction analysis (RERIs and TotRERIs).

Section G: Estimation of additive interactions between 3 risk factors from contingency tables

In this section, we present the calculation of interactions between 3 risk factors on the additive scale from contingency tables.

In the hypothetical example below, we present the risk *p* of the outcome D, in all potential combinations of presence or absence of the binary exposures X_1, X_2 and X_3

Table S1: Risk of outcome D by cross-classified exposure status, defined by presence/absence of the exposures X_1, X_2 and X_3

	**X_3=0**			**X_3=1**	
	**X_1=0**	**X_1=1**		**X_1=0**	**X_1=1**
**X_2=0**	$p_{X_1-X_2-X_3-} = 0.01$	$p_{X_1+X_2-X_3-} = 0.012$	**X_2=0**	$p_{X_1-X_2-X_3+} = 0.012$	$p_{X_1+X_2-X_3+} = 0.014$
**X_2=1**	$p_{X_1-X_2+X_3-} = 0.015$	$p_{X_1+X_2+X_3-} = 0.019$	**X_2=1**	$p_{X_1-X_2+X_3+} = 0.018$	$p_{X_1+X_2+X_3+} = 0.028$

From the table S1, we can directly calculate the risk ratios (RR) of developing the outcome D, when the corresponding risk factor are present or absent. Specifically,

$$RR_{\mathrm{X_1-X_2-X_3-}} = \frac{\mathrm{p_{X_1-X_2-X_3-}}}{\mathrm{p_{X_1-X_2-X_3-}}} = 1 \text{ (reference category)}$$

$$RR_{\mathrm{X_1+X_2-X_3-}} = \frac{\mathrm{p_{X_1\mp X_2-X_3-}}}{\mathrm{p_{X_1-X_2-X_3-}}} = 1.2$$

$$RR_{\mathrm{X_1-X_2+X_3-}} = \frac{\mathrm{p_{X_1-X_2+X_3-}}}{\mathrm{p_{X_1-X_2-X_3-}}} = 1.5$$

$$RR_{\mathrm{X_1-X_2-X_3+}} = \frac{\mathrm{p_{X_1-X_2-X_3+}}}{\mathrm{p_{X_1-X_2-X_3-}}} = 1.2$$

$$RR_{\mathrm{X_1+X_2+X_3-}} = \frac{\mathrm{p_{X_1+X_2+X_3-}}}{\mathrm{p_{X_1-X_2-X_3-}}} = 1.9$$

$$RR_{\mathrm{X_1+X_2-X_3+}} = \frac{\mathrm{p_{X_1+X_2-X_3+}}}{\mathrm{p_{X_1-X_2-X_3-}}} = 1.4$$

$$RR_{\mathrm{X_1-X_2+X_3+}} = \frac{\mathrm{p_{X_1-X_2+X_3+}}}{\mathrm{p_{X_1-X_2-X_3-}}} = 1.8$$

$$RR_{\mathrm{X_1+X_2+X_3+}} = \frac{\mathrm{p_{X_1+X_2+X_3+}}}{\mathrm{p_{X_1-X_2-X_3-}}} = 2.8$$

So, then it is straightforward to estimate all the potential 2-way and 3 way interactions, i.e.

$$RERI_2(\mathrm{X_1,X_2|X_3} = 0) = \left(\mathrm{RR_{X_1+X_2+X_3-}} - \mathrm{RR_{X_1-X_2-X_3-}}\right) - \left(\mathrm{RR_{X_1+X_2-X_3-}} - \mathrm{RR_{X_1-X_2-X_3-}}\right) - \left(\mathrm{RR_{X_1-X_2+X_3-}} - \mathrm{RR_{X_1-X_2-X_3-}}\right) = 0.2$$

$$RERI_2(\mathrm{X_1,X_3|X_2} = 0) = \left(\mathrm{RR_{X_1+X_2-X_3+}} - \mathrm{RR_{X_1-X_2-X_3-}}\right) - \left(\mathrm{RR_{X_1+X_2-X_3-}} - \mathrm{RR_{X_1-X_2-X_3-}}\right) - \left(\mathrm{RR_{X_1-X_2-X_3+}} - \mathrm{RR_{X_1-X_2-X_3-}}\right) = 0.1$$

$$RERI_2(\mathrm{X_2,X_3|X_1} = 0) = \left(\mathrm{RR_{X_1-X_2+X_3+}} - \mathrm{RR_{X_1-X_2-X_3-}}\right) - \left(\mathrm{RR_{X_1-X_2+X_3-}} - \mathrm{RR_{X_1-X_2-X_3-}}\right) - \left(\mathrm{RR_{X_1-X_2-X_3+}} - \mathrm{RR_{X_1-X_2-X_3-}}\right) = 0$$

$$\mathrm{RERI}_2(\mathrm{X}_1,\mathrm{X}_2|\mathrm{X}_3=1)=\frac{\left(\mathrm{RR}_{\mathrm{X}_1+\mathrm{X}_2+\mathrm{X}_3+}-\mathrm{RR}_{\mathrm{X}_1+\mathrm{X}_2-\mathrm{X}_3+}-\mathrm{RR}_{\mathrm{X}_1-\mathrm{X}_2+\mathrm{X}_3+}+\mathrm{RR}_{\mathrm{X}_1-\mathrm{X}_2-\mathrm{X}_3+}\right)}{\mathrm{RR}_{\mathrm{X}_1-\mathrm{X}_2-\mathrm{X}_3+}}=0.67$$

$$\mathrm{RERI}_2(\mathrm{X}_1,\mathrm{X}_3|\mathrm{X}_2=1)=\frac{\left(\mathrm{RR}_{\mathrm{X}_1+\mathrm{X}_2+\mathrm{X}_3+}-\mathrm{RR}_{\mathrm{X}_1+\mathrm{X}_2+\mathrm{X}_3-}-\mathrm{RR}_{\mathrm{X}_1-\mathrm{X}_2+\mathrm{X}_3+}+\mathrm{RR}_{\mathrm{X}_1-\mathrm{X}_2+\mathrm{X}_3-}\right)}{\mathrm{RR}_{\mathrm{X}_1-\mathrm{X}_2+\mathrm{X}_3-}}=0.58$$

$$\mathrm{RERI}_2(\mathrm{X}_2,\mathrm{X}_3|\mathrm{X}_l=1)=\frac{\left(\mathrm{RR}_{\mathrm{X}_1+\mathrm{X}_2+\mathrm{X}_3+}-\mathrm{RR}_{\mathrm{X}_1+\mathrm{X}_2+\mathrm{X}_3-}-\mathrm{RR}_{\mathrm{X}_1+\mathrm{X}_2-\mathrm{X}_3+}+\mathrm{RR}_{\mathrm{X}_1+\mathrm{X}_2-\mathrm{X}_3-}\right)}{\mathrm{RR}_{\mathrm{X}_1+\mathrm{X}_2-\mathrm{X}_3-}}=0.4$$

$$RERI_3(\mathrm{X}_1,\mathrm{X}_2,\mathrm{X}_3)=\mathrm{RR}_{\mathrm{X}_1+\mathrm{X}_2+\mathrm{X}_3+}-\mathrm{RR}_{\mathrm{X}_1+\mathrm{X}_2+\mathrm{X}_3-}-\mathrm{RR}_{\mathrm{X}_1+\mathrm{X}_2-\mathrm{X}_3+}-\mathrm{RR}_{\mathrm{X}_1-\mathrm{X}_2+\mathrm{X}_3+}+\mathrm{RR}_{\mathrm{X}_1+\mathrm{X}_2-\mathrm{X}_3-}+\mathrm{RR}_{\mathrm{X}_1-\mathrm{X}_2+\mathrm{X}_3-}+\mathrm{RR}_{\mathrm{X}_1-\mathrm{X}_2-\mathrm{X}_3+}-1=0.6$$

$$TotRERI_3(\mathrm{X}_1,\mathrm{X}_2,\mathrm{X}_3)=\mathrm{RR}_{\mathrm{X}_1+\mathrm{X}_2+\mathrm{X}_3+}-\mathrm{RR}_{\mathrm{X}_1+\mathrm{X}_2-\mathrm{X}_3-}-\mathrm{RR}_{\mathrm{X}_1-\mathrm{X}_2+\mathrm{X}_3-}-\mathrm{RR}_{\mathrm{X}_1-\mathrm{X}_2-\mathrm{X}_3+}+2=0.9$$

Section H: Simulation study

We conducted a small simulation study to demonstrate the accuracy of the proposed measures for additive interaction. The data generating mechanism and the values chosen for the simulations were informed by our motivating example. More specifically, we simulate event times (deaths) according to a Weibull model (λ=0.066, γ=2). As in the Greek-EPIC study, mortality rate in the simulated scenarios is around 8% and mean survival ~10 years. Given that the focus of the paper is on multi-way interactions, the simulation study focused on two main scenarios where:

We simulated the same log-hazard ratios with the Greek-EPIC study (i.e. beta coefficients from table 2, upper panel) in 2 different scenarios

Scenario 1: X_1, X_2, X_3 are simulated independently

Scenario 2: X_1, X_2, X_3 are correlated

The correlation parameters between X_1, X_2 and X_3 in scenario 2 were informed by our motivating example, i.e. $corr(X_1, X_2) = 0.027$, $corr(X_2, X_3) = -0.152$, $corr(X_1, X_3) = -0.008$

The simulations are presented in github (https://github.com/mkatsoulis82/Multi-way_interaction/blob/master/simulations.do)

The simulations assessed bias and confidence interval (CI) coverage across the different measures of additive interaction, including $TotRERI_3(X_1, X_2, X_3), RERI_3(X_1, X_2, X_3)$, $RERI_2(X_1, X_2|X_3 = 0), RERI_2(X_1, X_3|X_2 = 0), RERI_2(X_2, X_3|X_1 = 0), RERI_2(X_1, X_2|X_3 = 1)$, $RERI_2(X_1, X_3|X_2 = 1)$, and $RERI_2(X_2, X_3|X_1 = 1)$. These parameters were estimated by applying a Cox regression model to 1000 simulated datasets. The simulation results show negligible biases and CI coverage close to nominal levels (95%) across all the measures of additive interaction.

Table S2 – Parameter estimates, bias, percentage bias and confidence interval coverage for a scenario where the covariates X_1, X_2, X_3 are independent.

**Measures of additive interaction**	**True value (θ)**	**Estimate ($E(\hat{\theta})$)**	**Bias [$E(\hat{\theta}) - \theta$]**	**Bias (%)**	**CI coverage***
$TotRERI_3(X_1, X_2, X_3)$	1.179	1.189	0.010	0.9%	0.944
$RERI_3(X_1, X_2, X_3)$	1.970	2.005	0.036	1.8%	0.957
$RERI_2(X_1, X_2|X_3 = 0)$	-0.307	-0.315	-0.008	2.5%	0.958
$RERI_2(X_1, X_3|X_2 = 0)$	-0.224	-0.233	-0.009	4.0%	0.943
$RERI_2(X_2, X_3|X_1 = 0)$	-0.259	-0.268	-0.009	3.4%	0.957
$RERI_2(X_1, X_2|X_3 = 1)$	1.103	1.119	0.016	1.5%	0.944
$RERI_2(X_1, X_3|X_2 = 1)$	1.306	1.323	0.016	1.3%	0.950
$RERI_2(X_2, X_3|X_1 = 1)$	1.193	1.210	0.016	1.3%	0.953

*Confidence interval coverage nominal level is 0.95.

Table S3 – Parameter estimates, bias, percentage bias and confidence interval coverage for a scenario where the covariates X_1, X_2, X_3 are correlated†

**Measures of additive interaction**	**True value (θ)**	**Estimate $(\boldsymbol{E(\hat{\theta})})$**	**Bias** $[\boldsymbol{E(\hat{\theta}) - \theta}]$	**Bias (%)**	**CI coverage***
$TotRERI_3(X_1, X_2, X_3)$	1.179	1.203	0.025	2.1%	0.941
$RERI_3(X_1, X_2, X_3)$	1.970	2.007	0.037	1.9%	0.946
$RERI_2(X_1, X_2 \| X_3 = 0)$	-0.307	-0.313	-0.005	1.7%	0.957
$RERI_2(X_1, X_3 \| X_2 = 0)$	-0.224	-0.231	-0.007	3.1%	0.951
$RERI_2(X_2, X_3 \| X_1 = 0)$	-0.259	-0.260	-0.0004	0.2%	0.963
$RERI_2(X_1, X_2 \| X_3 = 1)$	1.103	1.125	0.022	2%	0.949
$RERI_2(X_1, X_3 \| X_2 = 1)$	1.306	1.327	0.021	1.6%	0.937
$RERI_2(X_2, X_3 \| X_1 = 1)$	1.193	1.127	0.024	2%	0.944

†$corr(X_1, X_2) = 0.027$, $corr(X_2, X_3) = -0.152$, $corr(X_1, X_3) = -0.008$,

*Confidence interval coverage nominal level is 0.95.
